# Supplementary material for: AXL confers cell migration and invasion by hijacking a PEAK1-regulated focal adhesion protein network
Source: Nat Commun. 2020 Jul 17;11:3586. doi: 10.1038/s41467-020-17415-x (PMC7368075; doi:10.1038/s41467-020-17415-x)
Supplement: Supplementary file 3 — Description of Additional Supplementary Files [file 41467_2020_17415_MOESM3_ESM.pdf]

Description of Additional Supplementary Files

File Name: Supplementary Data 1
Description: List of AXL phosphomodulated proteins upon GAS6 stimulation

File Name: Supplementary Data 2
Description: Table 2: Gene enrichment analysis of phosphomodulated proteins using gProfiler

File Name: Supplementary Data 3
Description: Table 3: List of NEDD9 preys obtained from BioID

File Name: Supplementary Data 4
Description: Table 4: Gene enrichment analysis of NEDD9 preys using gProfiler

File Name: Supplementary Data 5
Description: Table 5: List of PEAK1 wildtype and mutant preys obtained from BioID

File Name: Supplementary Data 6
Description: Table 6: Gene enrichment analysis of PEAK1 preys using gProfiler

File Name: Supplementary Movie 1
Description: Representative video of MDA-MB-231 cells expressing GFP-PAXILLIN treated with either DMSO or 1µM R428 and imaged live for 30min, Scale bar, 20µm.

File Name: Supplementary Movie 2
Description: Representative video of MDA-MB-231 cells expressing GFP-PAXILLIN serum starved and treated or not with GAS6 and imaged live for 30min, Scale bar, 20µm.

File Name: Supplementary Movie 3
Description: Representative video of MDA-MB-231 cells expressing GFP-PAXILLIN and Lifeact-RFP, transfected with 100nM of siCTRL or siPEAK1, and serum-starved and treated with or without GAS6 and imaged live for 30min, Scale bar, 20µm.
