# Supplementary material for: AXL confers cell migration and invasion by hijacking a PEAK1-regulated focal adhesion protein network
Source: Nat Commun. 2020 Jul 17;11:3586. doi: 10.1038/s41467-020-17415-x (PMC7368075; doi:10.1038/s41467-020-17415-x)
Supplement: Supplementary file 1 — Supplementary Information [file 41467_2020_17415_MOESM1_ESM.pdf]

Supplementary Information

AXL CONFERS CELL MIGRATION AND INVASION BY HIJACKING A PEAK1-REGULATED FOCAL ADHESION PROTEIN NETWORK

Abu-Thuraia et al.

a

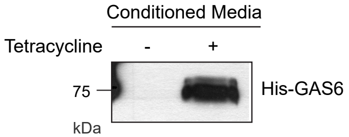

b

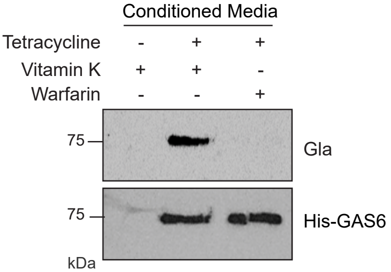

c

**Conditioned Media**	**GAS6 ng/mL**
-Tetracycline + Vit. K	0
+Tetracycline + Vit. K	260.8
+Tetracycline + Warfarin	110.9

d

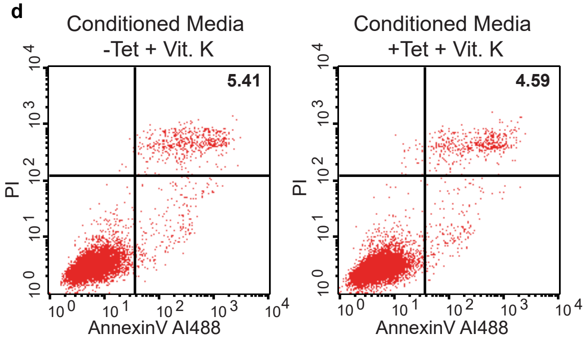

e

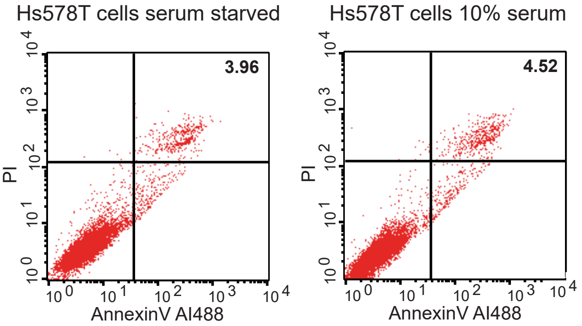

f

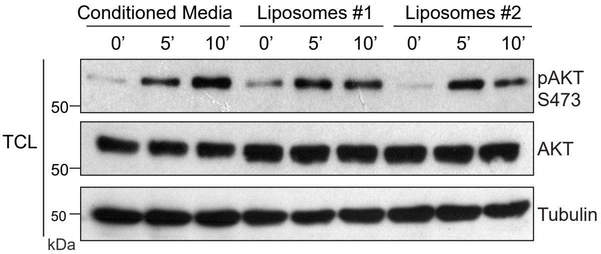

Supplementary Figure 1 Overview of the GAS6 conditioned media production and profiling. (**a**) Tetracycline-induced expression of GAS6-His in the media of Flp-InTM T-RExTM 293 cells. Cells were treated with Tetracycline and Vitamin K3 for 24 hours. (**b**) Flp-InTM T-RExTM 293 GAS6-His cells were treated with Tetracycline and Vitamin K3 and -/+ Warfarin for 24 hours. Media was assessed by western blotting for gamma-carboxylation state of GAS6, and the productions status of GAS6 using GLA and His antibody, respectively. (**c**) Using ELISA, GAS6 condition media upon the different treatments was quantified. (**d-e**) Scattor graphs of FACS quantified staining of apoptotic cells in GAS6 conditioned media (d) and -/+ serum grown Hs578T cells (e) using Annexin V and PI as readouts. The number in the upper right chamber represents the percentage of cells that are double positive for Annexin V and PI. (**f**) Lysates of serum starved Hs578T cells treated with GAS6 conditioned media or 10ug/mL (liposomes #1) or 100ug/mL (liposomes #2) of GAS6 coated liposomes for the indicated time. Levels of p-AKT and AKT were detected via western blotting.

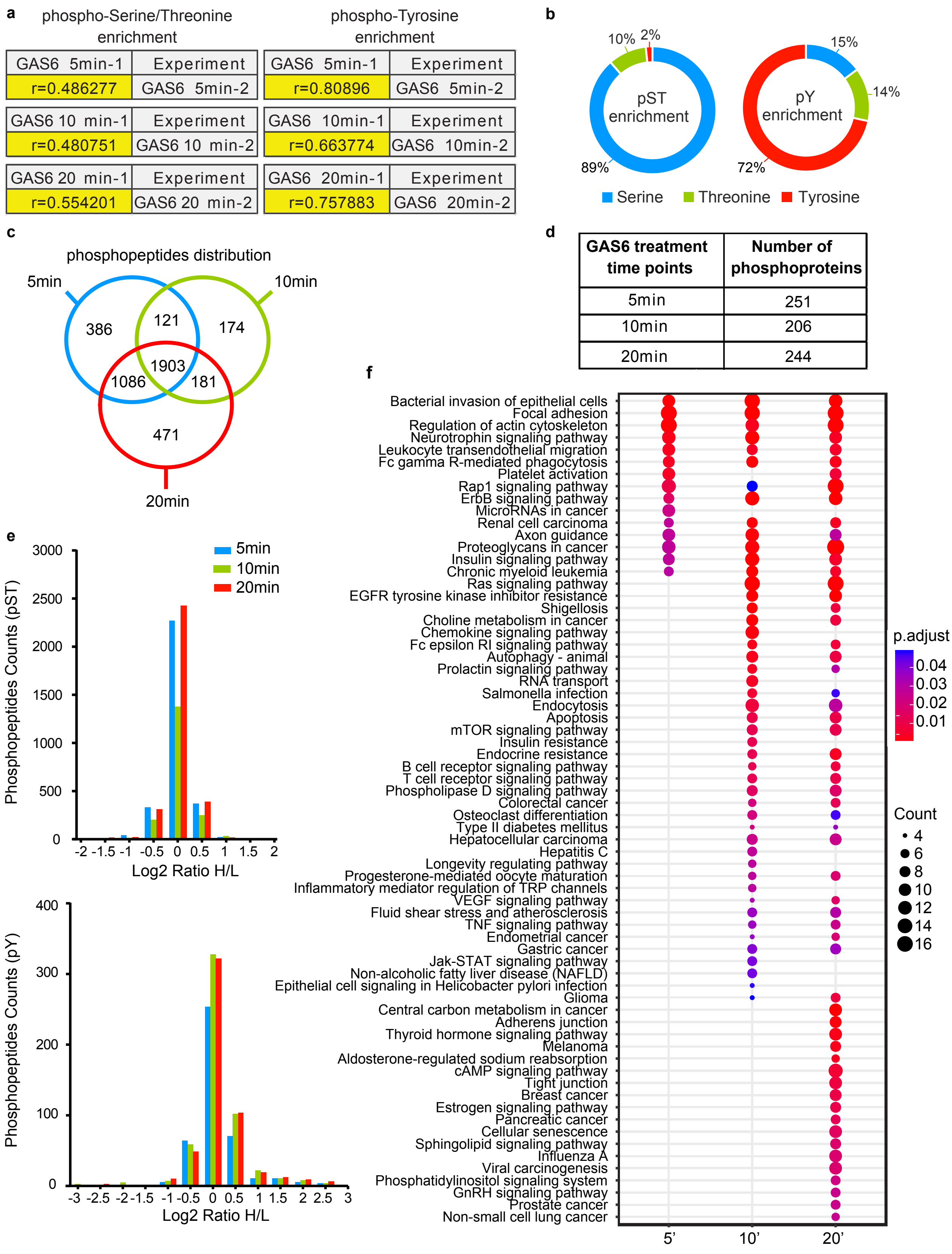
a
phospho-Serine/Threonine enrichment
GAS6 5min-1
Experiment
r=0.486277
GAS6 5min-2
GAS6 10 min-1
Experiment
r=0.480751
GAS6 10 min-2
GAS6 20 min-1
Experiment
r=0.554201
GAS6 20 min-2
phospho-Tyrosine enrichment
GAS6 5min-1
Experiment
r=0.80896
GAS6 5min-2
GAS6 10min-1
Experiment
r=0.663774
GAS6 10min-2
GAS6 20min-1
Experiment
r=0.757883
GAS6 20min-2
b
10%
2%
89%
pST enrichment
15%
14%
72%
pY enrichment
Serine
Threonine
Tyrosine
c
phosphopeptides distribution
5min
10min
20min
386
121
174
1903
1086
181
471
d
GAS6 treatment time points
Number of phosphoproteins
5min
251
10min
206
20min
244
e
5min
10min
20min
Phosphopeptides Counts (pST)
3000
2500
2000
1500
1000
500
0
-2 -1.5 -1 -0.5 0 0.5 1 1.5 2
Log2 Ratio H/L
Phosphopeptides Counts (pY)
400
300
200
100
0
-3 -2.5 -2 -1.5 -1 -0.5 0 0.5 1 1.5 2 2.5 3
Log2 Ratio H/L
f
Bacterial invasion of epithelial cells
Focal adhesion
Regulation of actin cytoskeleton
Neurotrophin signaling pathway
Leukocyte transendothelial migration
Fc gamma R-mediated phagocytosis
Platelet activation
Rap1 signaling pathway
ErbB signaling pathway
MicroRNAs in cancer
Renal cell carcinoma
Axon guidance
Proteoglycans in cancer
Insulin signaling pathway
Chronic myeloid leukemia
Ras signaling pathway
EGFR tyrosine kinase inhibitor resistance
Shigellosis
Choline metabolism in cancer
Chemokine signaling pathway
Fc epsilon RI signaling pathway
Autophagy - animal
Prolactin signaling pathway
RNA transport
Salmonella infection
Endocytosis
Apoptosis
mTOR signaling pathway
Insulin resistance
Endocrine resistance
B cell receptor signaling pathway
T cell receptor signaling pathway
Phospholipase D signaling pathway
Colorectal cancer
Osteoclast differentiation
Type II diabetes mellitus
Hepatocellular carcinoma
Hepatitis C
Longevity regulating pathway
Progesterone-mediated oocyte maturation
Inflammatory mediator regulation of TRP channels
VEGF signaling pathway
Fluid shear stress and atherosclerosis
TNF signaling pathway
Endometrial cancer
Gastric cancer
Jak-STAT signaling pathway
Non-alcoholic fatty liver disease (NAFLD)
Epithelial cell signaling in Helicobacter pylori infection
Glioma
Central carbon metabolism in cancer
Adherens junction
Thyroid hormone signaling pathway
Melanoma
Aldosterone-regulated sodium reabsorption
cAMP signaling pathway
Tight junction
Breast cancer
Estrogen signaling pathway
Pancreatic cancer
Cellular senescence
Sphingolipid signaling pathway
Influenza A
Viral carcinogenesis
Phosphatidylinositol signaling system
GnRH signaling pathway
Prostate cancer
Non-small cell lung cancer
5'
10'
20'
p.adjust
0.04
0.03
0.02
0.01
Count
4
6
8
10
12
14
16

Supplementary Figure 2 Overview of the GAS6 phosphoproteome profiling. (**a**) Pearson Correlation Coefficient (r) between biological replicates of phospho-S/T and phospho-Y peptides enriched from GAS6-stimulated cells during 5, 10 and 20 min. (**b**) Pie charts indicating the percentage of Serine, Threonine and Tyrosine phosphorylation sites identified in GAS6 treatment following pS/T and pY peptides enrichments. (**c**) Venn diagram showing the phosphopeptides distributions and overlaps between the three different time points of GAS6 stimulation. (**d**) Enumeration table of phosphoproteins identified at each GAS6 stimulation time point. (**e**) Log_2 ratio H/L distribution of pS/T or pY enriched peptides at 5-, 10- and 20- min. (**f**) Dot plot representation of significantly enriched KEGG pathways of GAS6 regulated phosphoproteins at the three different time points of stimulation. Circle sizes represent the number of regulated phosphoproteins associated to the specific pathway and the color of the circle represents the significant adjusted p-value.

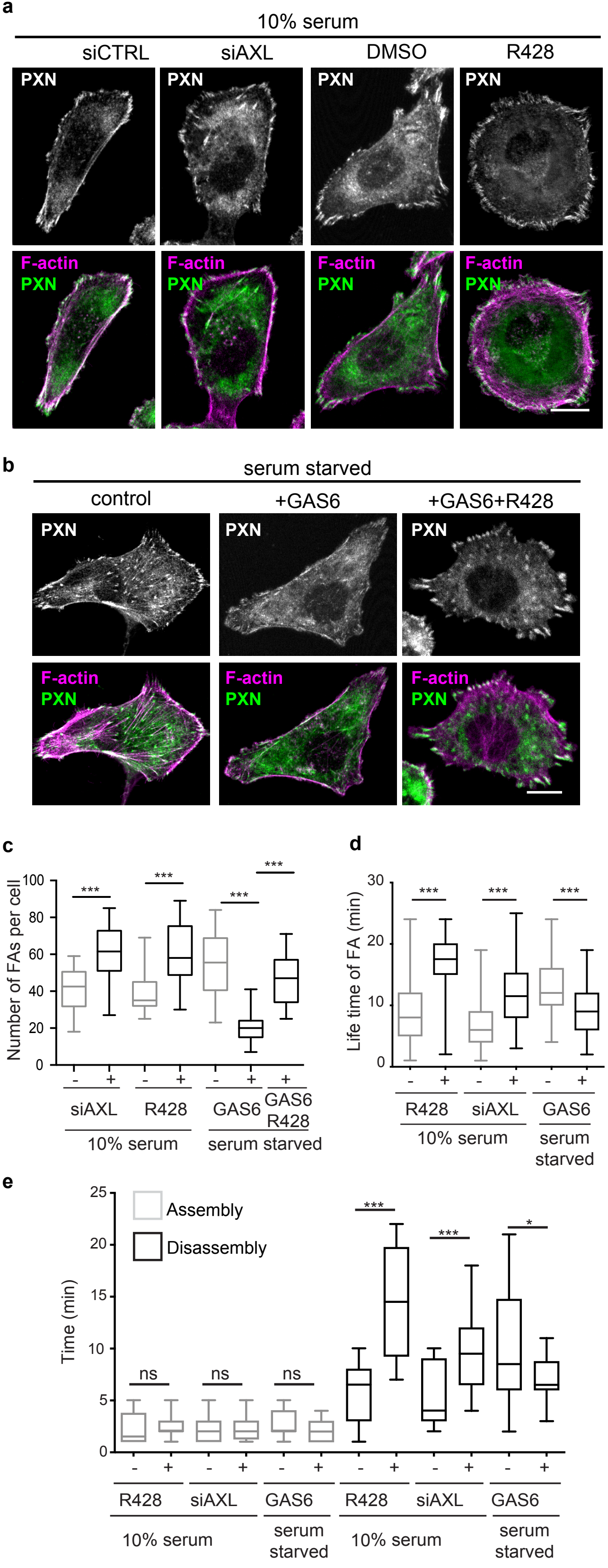
a
10% serum
siCTRL
siAXL
DMSO
R428
PXN
F-actin
PXN
b
serum starved
control
+GAS6
+GAS6+R428
PXN
F-actin
PXN
c
Number of FAs per cell
***
siAXL
R428
GAS6
GAS6 R428
10% serum
serum starved
d
Life time of FA (min)
***
R428
siAXL
GAS6
10% serum
serum starved
e
Assembly
Disassembly
Time (min)
ns
***
*
R428
siAXL
GAS6
10% serum
serum starved

Supplementary Figure 3 AXL modulates FA turnover in TNBC cells (**a-b**) AXL regulates number of FAs. Representative confocal images of MDA-MB-231 cells either treated with DMSO or 1µM R428, transfected with 100nM of siCTRL or siAXL (a), serum-starved and treated with GAS6 for 20min or serum starved and treated with GAS6 and R428 (b). Cells were stained for PXN (green) and Phalloidin (magenta), Scale bar, 10µm. (**c**) Quantification of the FA number per cell depicted in (a-b). *** $P < 0.000001$ (n=3 experiments with 30 cells per condition). (**d-e**) Quantification of FA life time (d) and their assembly and disassembly time (e) of Hs578T cells transfected with GFP-PXN either treated with DMSO or 1µM R428, transfected with 100nM of siCTRL or siAXL or serum-starved and treated with GAS6 for 20min. *** $P < 0.000001$ (d); **** $P < 0.000001$ (e); *** $P = 0.000074$ (e); * $P = 0.0149$ (e) (n=3 experiments with 90 FAs followed per condition). Data are represented as boxplots where the middle line is the median, the lower and upper hinges correspond to the first and third quartiles, the upper whisker extends from the hinge to the largest value and the lower whisker extends from the hinge to the smallest value. Two-tailed Mann–Whitney test was used to calculate p-value.

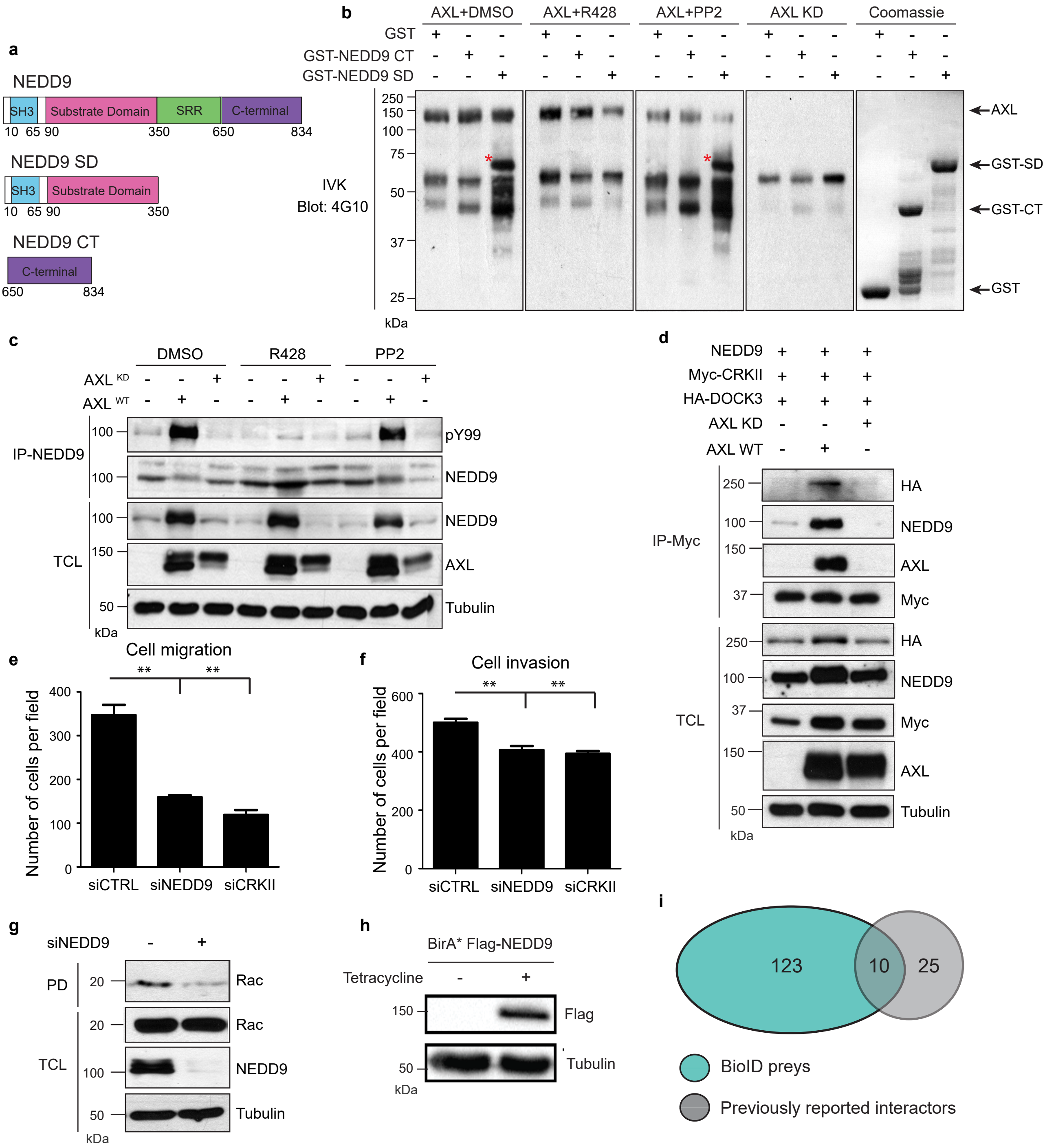
a
NEDD9
SH3
Substrate Domain
SRR
C-terminal
10 65 90 350 650 834
NEDD9 SD
SH3
Substrate Domain
10 65 90 350
NEDD9 CT
C-terminal
650 834
b
AXL+DMSO
AXL+R428
AXL+PP2
AXL KD
Coomassie
GST
GST-NEDD9 CT
GST-NEDD9 SD
IVK
Blot: 4G10
250
150
100
75
50
37
25
kDa
AXL
GST-SD
GST-CT
GST
c
DMSO
R428
PP2
AXL KD
AXL WT
IP-NEDD9
TCL
pY99
NEDD9
NEDD9
AXL
Tubulin
100
100
100
150
50
kDa
d
NEDD9
Myc-CRKII
HA-DOCK3
AXL KD
AXL WT
IP-Myc
TCL
HA
NEDD9
AXL
Myc
HA
NEDD9
Myc
AXL
Tubulin
250
100
150
37
250
100
37
150
50
kDa
e
Cell migration
Number of cells per field
400
300
200
100
0
**
**
siCTRL
siNEDD9
siCRKII
f
Cell invasion
Number of cells per field
600
400
200
0
**
**
siCTRL
siNEDD9
siCRKII
g
siNEDD9
PD
TCL
Rac
Rac
NEDD9
Tubulin
20
20
100
50
kDa
h
BirA* Flag-NEDD9
Tetracycline
Flag
Tubulin
150
50
kDa
i
123
10
25
BioID preys
Previously reported interactors

Supplementary Figure 4 AXL modulates NEDD9 phosphorylation levels *in vitro* and its complex formation. (**a**) NEDD9 constructs used for the in vitro kinase assay. (**b**) AXL phosphorylates NEDD9 *in vitro.* Lysates of 293T cells expressing AXL WT or KD (kinase dead) were used for anti-AXL immunoprecipitation. AXL immunoprecipitates were subjected to an in vitro kinase assay using the constructs shown in (a) as substrates, in the presence of either AXL inhibitor (1µM R428) or Src inhibitor (5µM PP2). NEDD9 phosphorylation levels were detected via western blotting. (**c**) AXL phosphorylates NEDD9 in 293T cells. Lysates of 293T cells expressing indicated plasmids and treated with either DMSO, R428 or PP2 were subjected to anti-NEDD9 immunoprecipitation. Co-immunoprecipitates were detected via western blotting. (**d**) AXL kinase activity regulates NEDD9 complex formation with CRKII/DOCK3. Lysates of 293T cells expressing indicated plasmids were subjected to anti-Myc CRKII immunoprecipitation. Co-immunoprecipitates were detected via western blotting. (**e-f**) Boyden migration (e) and invasion (f) assays performed with MDA-MB-231 cells transfected with 100nM of siCTRL, siNEDD9 or siCRKII using Matrigel as a matrix in the invasion assay. Number of cells migrated were counted via DAPI staining of the membrane. **** $P < 0.00001$ (n=3, 10 fields used at 10X per condition per experiment). (**g**) RAC1 activation levels are NEDD9 regulated in TNBC cells. MDA-MB-231 cells transfected with 100nM of siCTRL or siNEDD9 were subjected to a RAC activation Assay (GST-PAK1 PBD pulldown). RAC activation levels were detected via western blotting. (**h**) Tetracycline-induced expression of BirA-Flag-NEDD9 in Flp-InTM T-RExTM 293 cells. Cells were treated with Tetracycline for 24 hours. (**i**) A venn diagram comparing the number of NEDD9 interactors detected in the BioID screen versus the previously reported ones. Data were analyzed from three experiments and expressed as mean ± s.e.m. Two-tailed Mann–Whitney test was used (e, f).

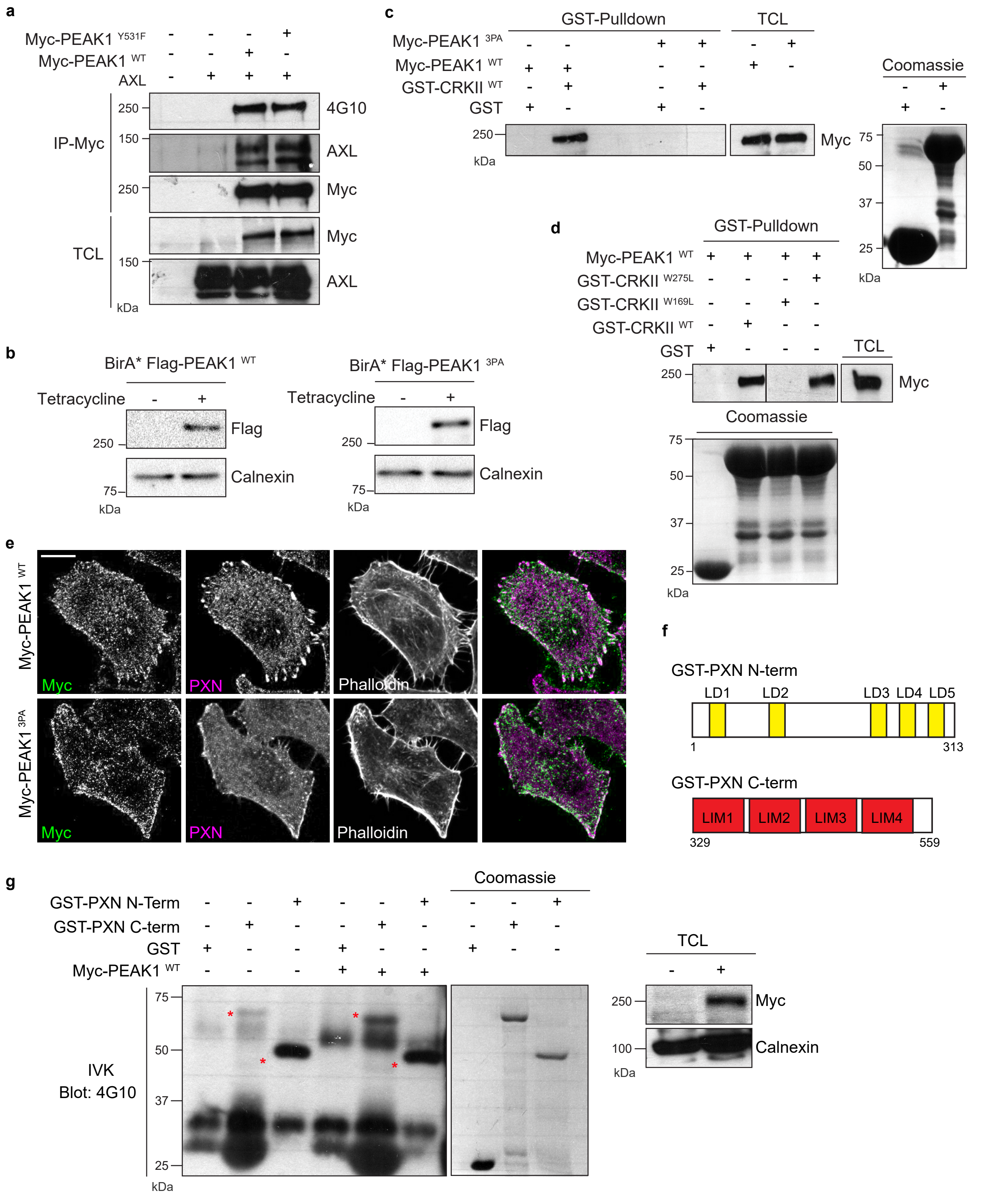

a
Myc-PEAK1 Y531F
Myc-PEAK1 WT
AXL
IP-Myc
TCL
4G10
AXL
Myc
Myc
AXL
250
150
250
150
kDa
b
BirA* Flag-PEAK1 WT
BirA* Flag-PEAK1 3PA
Tetracycline
Flag
Calnexin
250
75
kDa
c
GST-Pulldown
TCL
Myc-PEAK1 3PA
Myc-PEAK1 WT
GST-CRKII WT
GST
Myc
Coomassie
250
kDa
75
50
37
25
d
GST-Pulldown
Myc-PEAK1 WT
GST-CRKII W275L
GST-CRKII W169L
GST-CRKII WT
GST
TCL
Myc
Coomassie
250
75
50
37
25
kDa
e
Myc-PEAK1 WT
Myc-PEAK1 3PA
Myc
PXN
Phalloidin
f
GST-PXN N-term
LD1
LD2
LD3
LD4
LD5
1
313
GST-PXN C-term
LIM1
LIM2
LIM3
LIM4
329
559
g
Coomassie
GST-PXN N-Term
GST-PXN C-term
GST
Myc-PEAK1 WT
IVK
Blot: 4G10
75
50
37
25
kDa
TCL
Myc
Calnexin
250
100

Supplementary Figure 5 PEAK1 interacts with CRKII and mediates PXN phosphorylation. (**a**) Lysates of 293T cells expressing the indicated plasmids were used for anti-Myc immunoprecipitation. The levels of Myc-PEAK1 phosphorylation and AXL are detected via western blotting. (**b**) Tetracycline-induced expression of BirA-Flag-PEAK1 and BirA-Flag-PEAK1 3PA in Flp-InTM T-RExTM 293 cells. Cells were treated with Tetracycline for 24 hours. (**c**) Lysates of 293T cells expressing Myc-PEAK1 WT or 3PA mutant were used for GST-CRKII pulldown. Levels of Myc-PEAK1 is detected via western blotting. (**d**) PEAK1 binds CRKII middle SH3 domain. Lysates of 293T cells expressing Myc-PEAK1 were used for GST-CRKII pulldown using the different GST-CRKII constructs. Levels of Myc-PEAK1 is detected via western blotting. (**e**) Representative confocal images of HELA cells transfected with Myc-PEAK1 WT or 3PA mutant. Fixed cells were permeabilized and stained for Myc (green), PXN (red) and Phalloidin (grey). (n=3 experiments, 15 cells per condition per experiment). Scale bar, 20μm. (**f**) Schematic of GST-PXN domains. (**g**) PEAK1 induces phosphorylation of PXN *in vitro.* Lysates of 293T cells expressing or not Myc-PEAK1 were used for anti-Myc immunoprecipitation. Myc-PEAK1 immunoprecipitates were subjected to an invitro kinase assay using the GST constructs indicated as substrates, shown in Coomassie staining. PXN phosphorylation levels were detected via western blotting. The band of interest that corresponds to each substrate is indicated with a *.

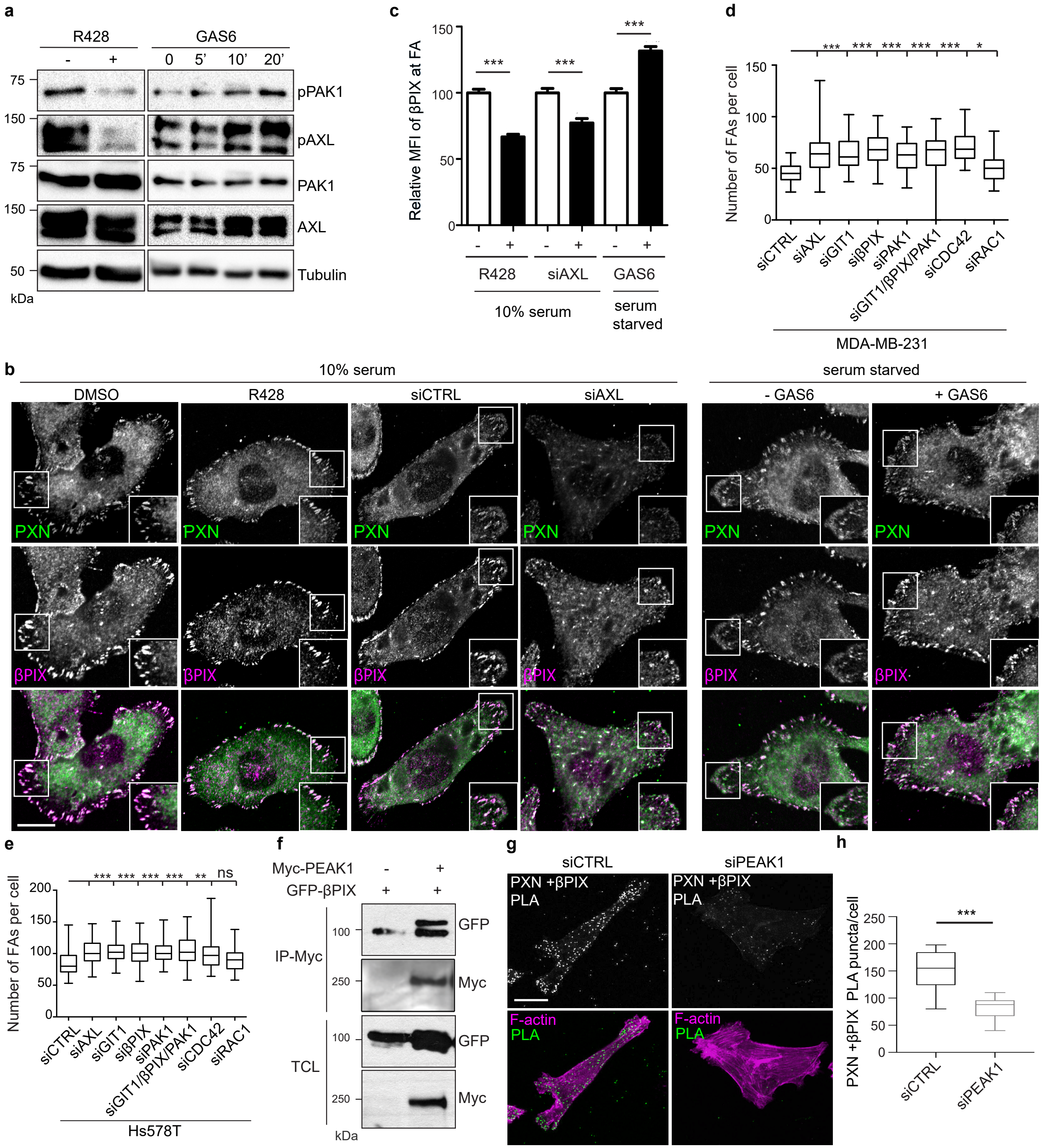

a
R428
GAS6
-
+
0
5'
10'
20'
75
150
75
150
50
kDa
pPAK1
pAXL
PAK1
AXL
Tubulin
c
Relative MFI of βPIX at FA
150
100
50
0
***
***
***
R428
siAXL
GAS6
10% serum
serum starved
d
Number of FAs per cell
150
100
50
0
*** *** *** *** *** *
siCTRL
siAXL
siGIT1
siβPIX
siPAK1
siGIT1/βPIX/PAK1
siCDC42
siRAC1
MDA-MB-231
b
10% serum
serum starved
DMSO
R428
siCTRL
siAXL
- GAS6
+ GAS6
PXN
βPIX
e
Number of FAs per cell
200
150
100
50
0
*** *** *** *** ** ns
siCTRL
siAXL
siGIT1
siβPIX
siPAK1
siGIT1/βPIX/PAK1
siCDC42
siRAC1
Hs578T
f
Myc-PEAK1
GFP-βPIX
IP-Myc
TCL
100
250
100
250
GFP
Myc
GFP
Myc
kDa
g
siCTRL
siPEAK1
PXN +βPIX
PLA
F-actin
PLA
h
PXN +βPIX PLA puncta/cell
250
200
150
100
50
0
***
siCTRL
siPEAK1

Supplementary Figure 6 AXL/PEAK1 modulate βPIX/GIT1/PAK complex. (**a**) Modulation of PAK1 phosphorylation by AXL activation or inhibition. Lysates of cells either treated with 1µM R428 for one hour or serum starved and treated with GAS6 for indicated time were analyzed with the indicated antibodies. (**b**) βPIX recruitment to FA is AXL modulated. Representative confocal images of Hs578T cells plated on coverslips and either treated with DMSO or 1µM R428, transfected with 100nM of siCTRL or siAXL or serum-starved and treated with GAS6 for 20min. Cells were then fixed, permeabilized and stained for PXN (green) and βPIX (magents). Boxes are used to depict the location of the zoomed images. Scale bar, 20µm. (**c**) Quantification of mean fluorescence intensity (MFI) of βPIX at PXN-FA shown in (b). **** $P < 0.00001$. (**d-e**) Number of FAs is modulated by GIT1/βPIX/PAK1/CDC42/RAC1 expression levels. Quantification of the FA number per cell in Hs578T (d) or MDA-MB-231 (e) cells transfected with the indicated siRNA. ** $P < 0.005$ *** $P \leq 0.0001$ (n=3 experiments with 30 cells per condition). (**f**) PEAK1 interacts with βPIX. Lysates of 293T cells expressing the indicated plasmids were used for anti-Myc immunoprecipitation. Levels of GFP-βPIX is detected via western blotting. (**g**) PEAK1 regulates βPIX localization at FA sites Representative confocal images of MDA-MB-231 cells transfected with either 100nM siCTRL or siPEAK1. PLA kit was used to analyze localization of βPIX at PXN-FAs. Scale bar, 20µm. (**h**) Quantifications of the number of PLA puncta per cell per condition. *** $P < 0.0001$. (n=3 experiments, 15 cells per condition per experiment). Data are represented as boxplots where the middle line is the median, the lower and upper hinges correspond to the first and third quartiles, the upper whisker extends from the hinge to the largest value and the lower whisker extends from the hinge to the smallest value (c-e, h). Two-tailed Mann–Whitney test was used to calculate p-value.

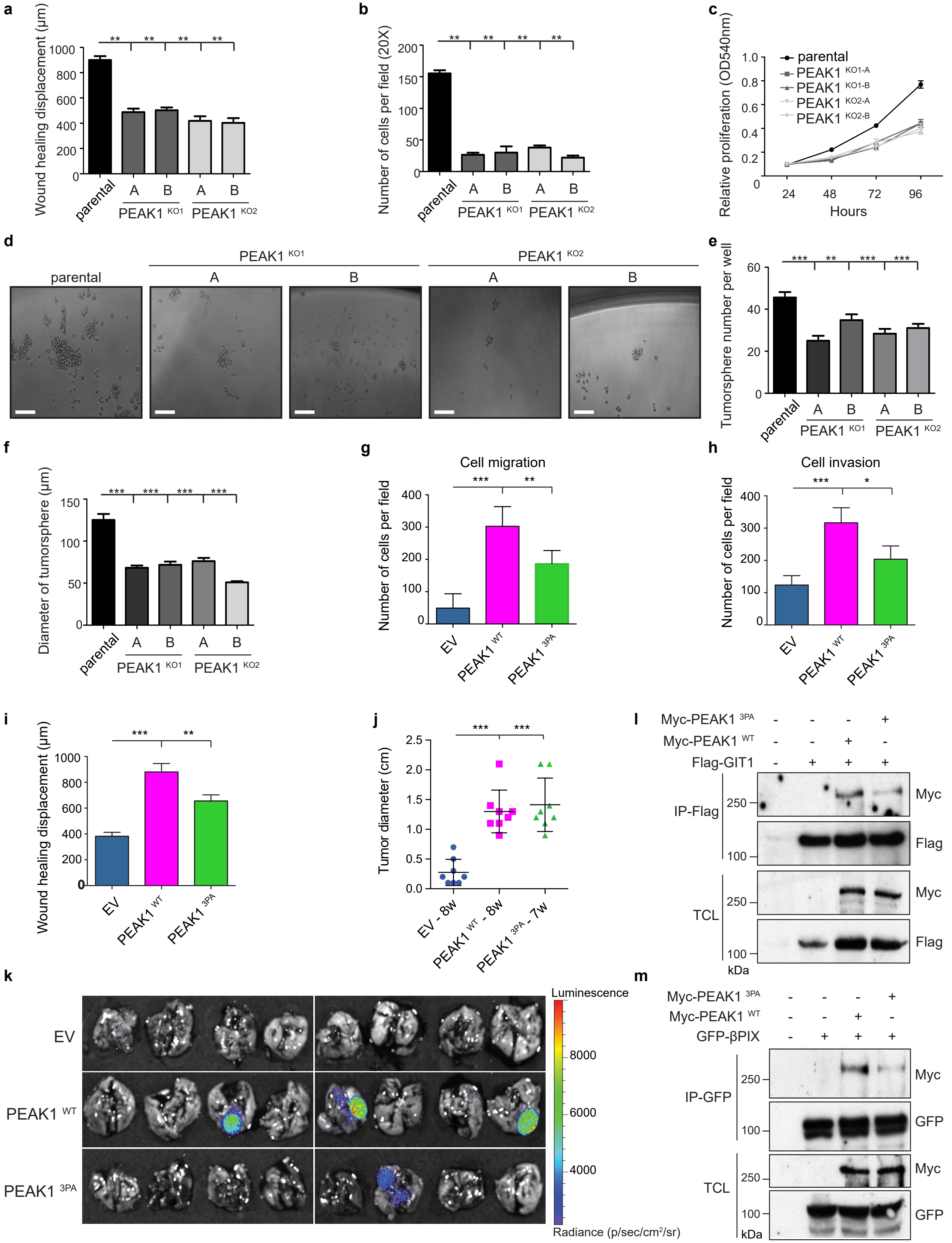

a
Wound healing displacement (μm)
parental
A
B
A
B
PEAK1 KO1
PEAK1 KO2
b
Number of cells per field (20X)
c
Relative proliferation (OD540nm)
parental
PEAK1 KO1-A
PEAK1 KO1-B
PEAK1 KO2-A
PEAK1 KO2-B
Hours
d
parental
PEAK1 KO1
PEAK1 KO2
e
Tumorsphere number per well
f
Diameter of tumorsphere (μm)
g
Cell migration
Number of cells per field
EV
PEAK1 WT
PEAK1 3PA
h
Cell invasion
Number of cells per field
i
Wound healing displacement (μm)
j
Tumor diameter (cm)
EV - 8w
PEAK1 WT - 8w
PEAK1 3PA - 7w
k
EV
PEAK1 WT
PEAK1 3PA
Luminescence
Radiance (p/sec/cm²/sr)
l
Myc-PEAK1 3PA
Myc-PEAK1 WT
Flag-GIT1
IP-Flag
TCL
Myc
Flag
kDa
m
Myc-PEAK1 3PA
Myc-PEAK1 WT
GFP-βPIX
IP-GFP
TCL
Myc
GFP
kDa

Supplementary Figure 7 PEAK1 is required for wound healing, proliferation and tumorsphere formation *in vitro*. (**a**) Quantifications of MDA-MB-231 CRISPR WT or PEAK1 KO1 or KO2 clones displacement in a wound healing assay. *** $P = 0.0002$ (n=3, 4 fields used at 10X per condition per experiment). (**b**) Boyden invasion assay performed with MDA-MB-231 CRISPR WT or PEAK1 KO1 or KO2 clones using Matrigel as a matrix. Number of cells invaded were counted via DAPI staining of the membrane. **** $P < 0.0001$ (n=3, 10 fields used at 10X per condition per experiment). (**c**) MTT proliferation assay done in MDA-MB-231 CRISPR WT or PEAK1 KO1 or KO2 clones. N= 3 independent experiments with 5 technical replicates for each cell type per day. (**d**) Representative microscopy images of tumorsphere formation in MDA-MB-231 CRISPR WT or PEAK1 KO1 or KO2 clones after 7 days. Scale bar, 60µm. (**e-f**) Quantifications of the tumorsphere number (e) and size of the tumorsphere (f) in conditions shown in (d). ** $P = 0.0083$, *** $P = 0.0002$, **** $P < 0.0001$ (n=3, 10 fields used at 40X per condition per experiment). (**g-h**) Boyden migration (g) and invasion (h) assay performed with MDA-MB-231 PEAK1 KO rescued with either EV, PEAK1 WT or PEAK1 3PA using Matrigel as a matrix for invasion assay. Number of cells migrated (g) or invaded (h) were counted via DAPI staining of the membrane. **** $P < 0.0001$ (n=3, 10 fields used at 10X per condition per experiment). (**i**) Quantifications of MDA-MB-231 PEAK1 KO rescued with either EV, PEAK1 WT or PEAK1 3PA displacement in a wound healing assay. *** $P = 0.0002$ (n=3, 4 fields used at 10X per condition per experiment). (**j**) Tumors were excised out of the mice at the indicated time post-injection and measured for their diameter for the three different conditions. N=8 for each group *** $P < 0.0001$. (**k**) Representative bioluminescent lung images of mice shown in Fig 10b. For the lungs of the mice bearing MDA-MB-231-Luc PEAK1 knockouts rescued with EV, PEAK1 WT or PEAK1 3PA tumors, lungs were dissected once the tumors reached the same size. (**l**) Lysates of 293T cells expressing the indicated plasmids were used for anti-Flag immunoprecipitation. Levels of Myc-PEAK1 is detected via western blotting. (**m**) Lysates of 293T cells expressing the indicated plasmids were used for anti-GFP immunoprecipitation. Levels of Myc-PEAK1 is detected via western blotting. Data were analyzed from three experiments and expressed as mean ± s.e.m (a, b, g-i, j). Data are represented as boxplots where the middle line is the median, the lower and upper hinges correspond to the first and third quartiles, the upper whisker extends from the hinge to the largest value and the lower whisker extends from the hinge to the smallest value (e, f). Two-tailed Mann–Whitney test was used to calculate all p-values.

Supplementary Figure 8

Raw Western blot scans

Fig 1b

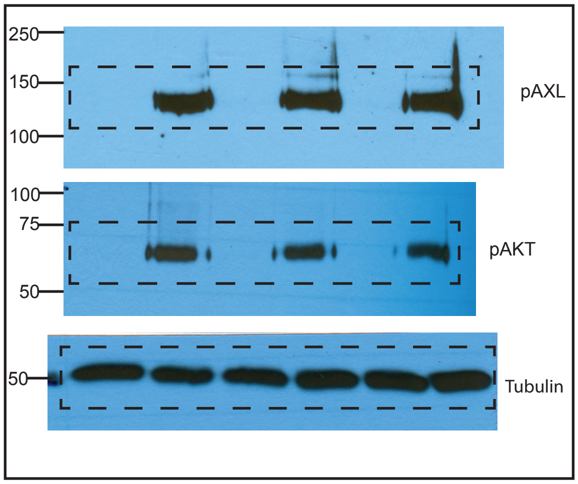
250
150
100
pAXL
100
75
50
pAKT
50
Tubulin

Fig 1e

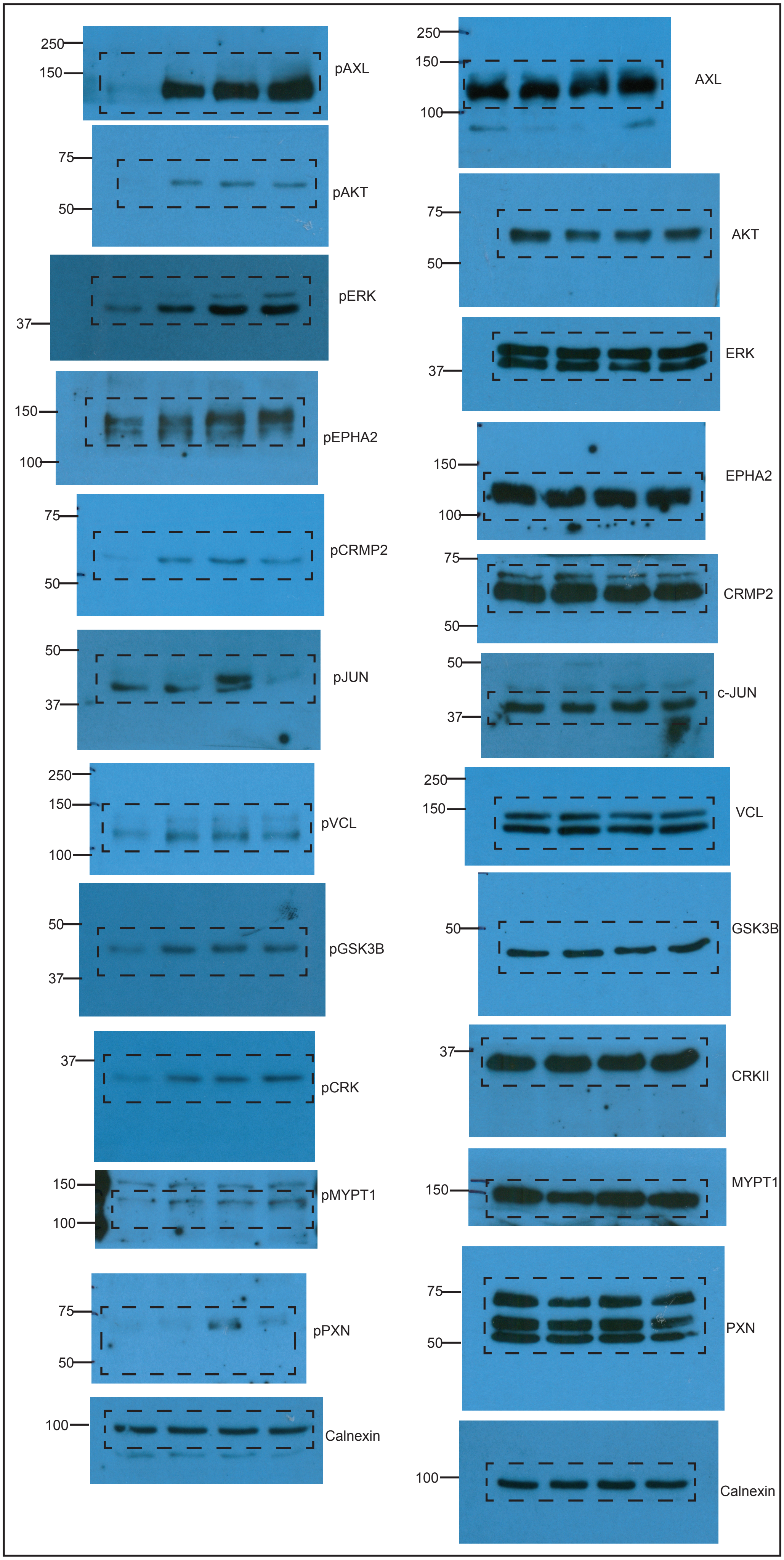
250
150
pAXL
75
50
pAKT
37
pERK
150
100
pEPHA2
75
50
pCRMP2
50
37
pJUN
250
150
100
pVCL
50
37
pGSK3B
37
pCRK
150
100
pMYPT1
75
50
pPXN
100
Calnexin
250
150
100
AXL
75
50
AKT
37
ERK
150
100
EPHA2
75
50
CRMP2
50
37
c-JUN
250
150
VCL
50
GSK3B
37
CRKII
150
MYPT1
75
50
PXN
100
Calnexin

Fig 4a

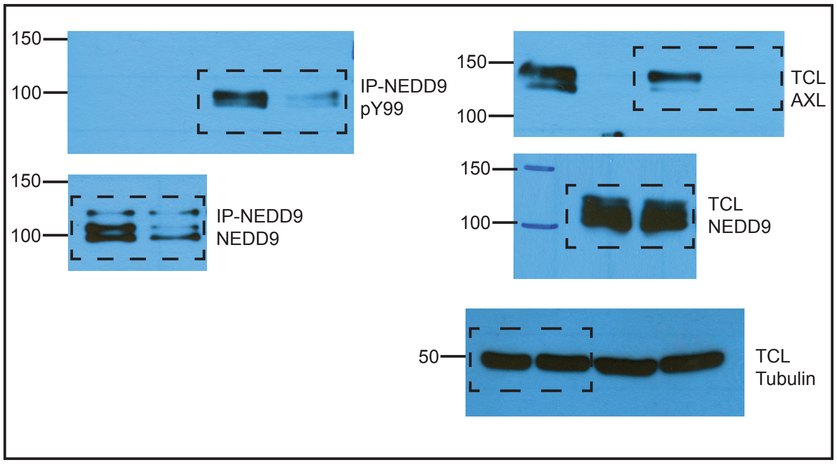

Fig 4b

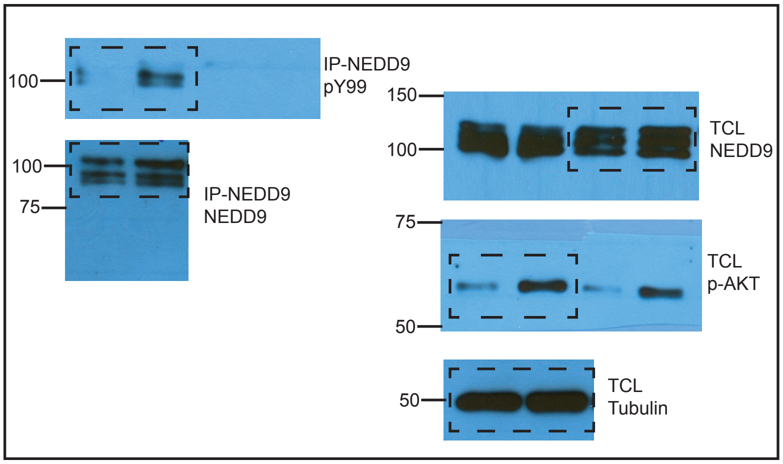

Fig 4c

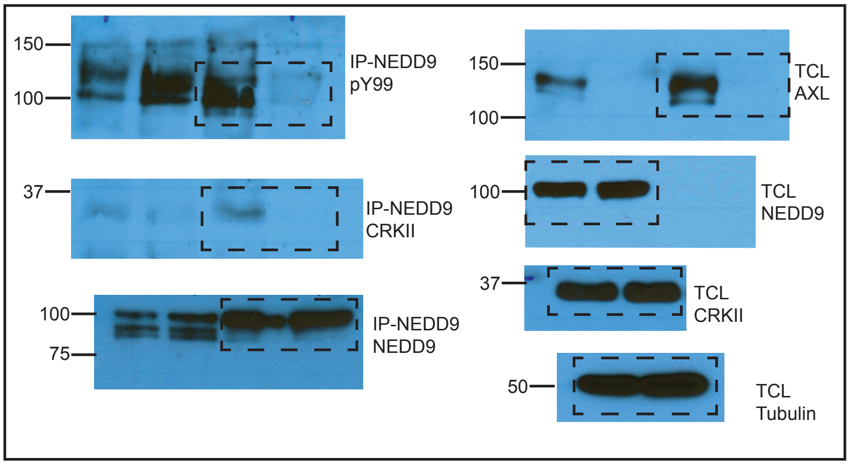

Fig 4f

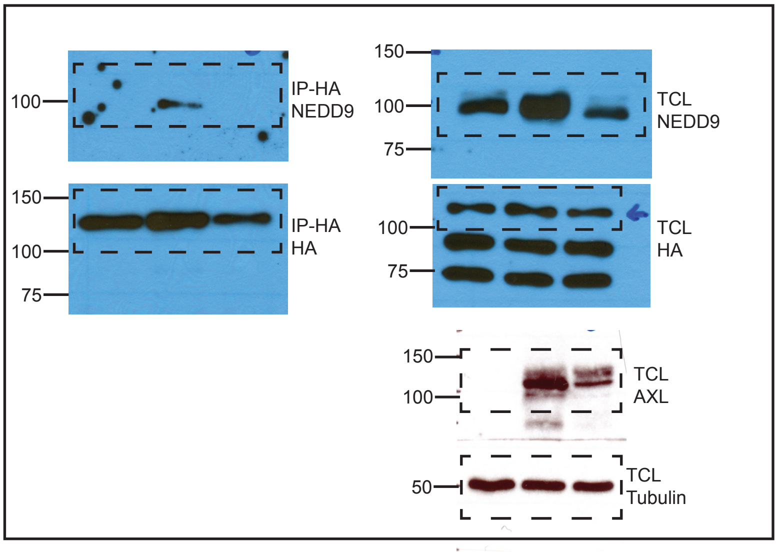

Fig 5b

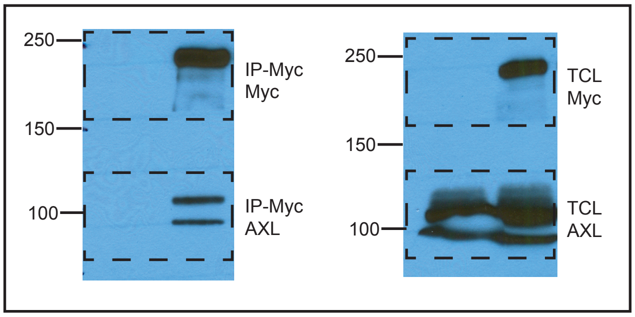
250
150
100
IP-Myc
Myc
IP-Myc
AXL
250
150
100
TCL
Myc
TCL
AXL

Fig 5c

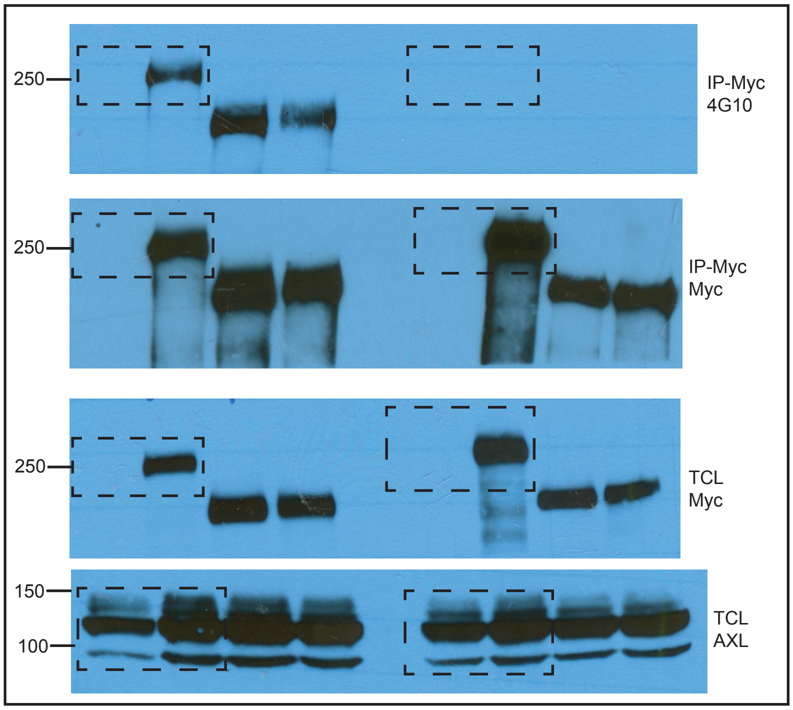
250
IP-Myc
4G10
250
IP-Myc
Myc
250
TCL
Myc
150
100
TCL
AXL

Fig 6b

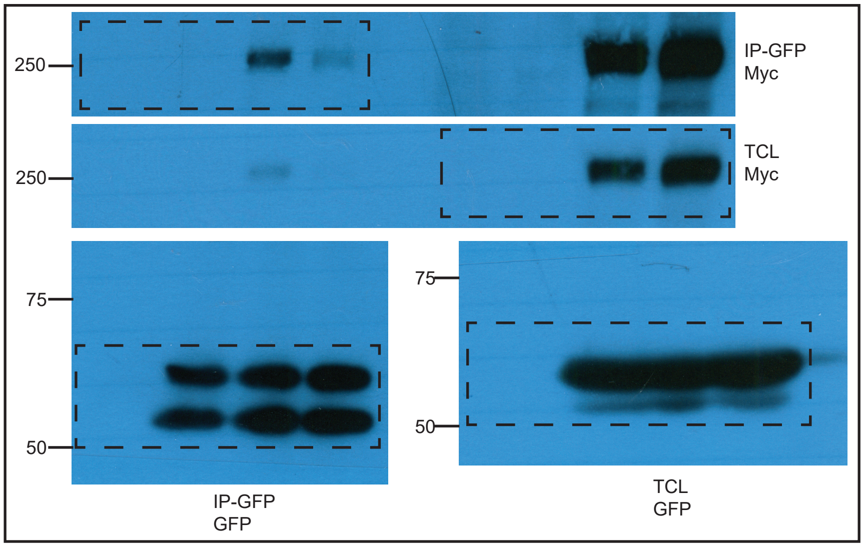

Fig 6c

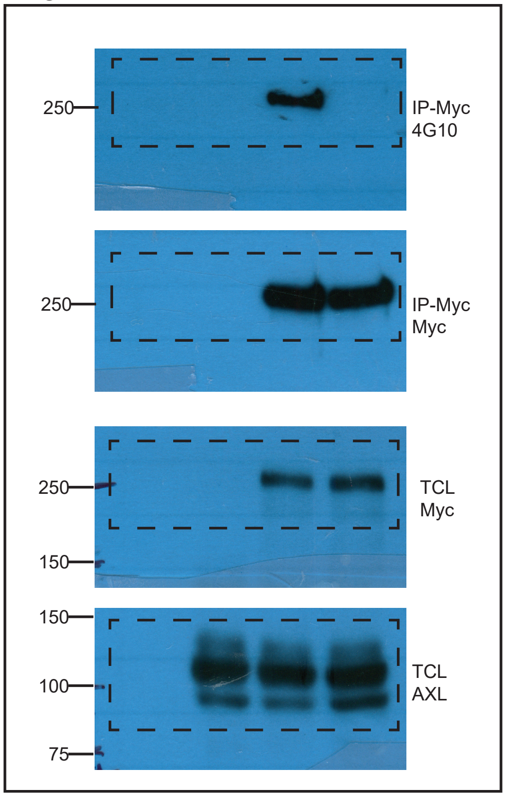

Fig 6d

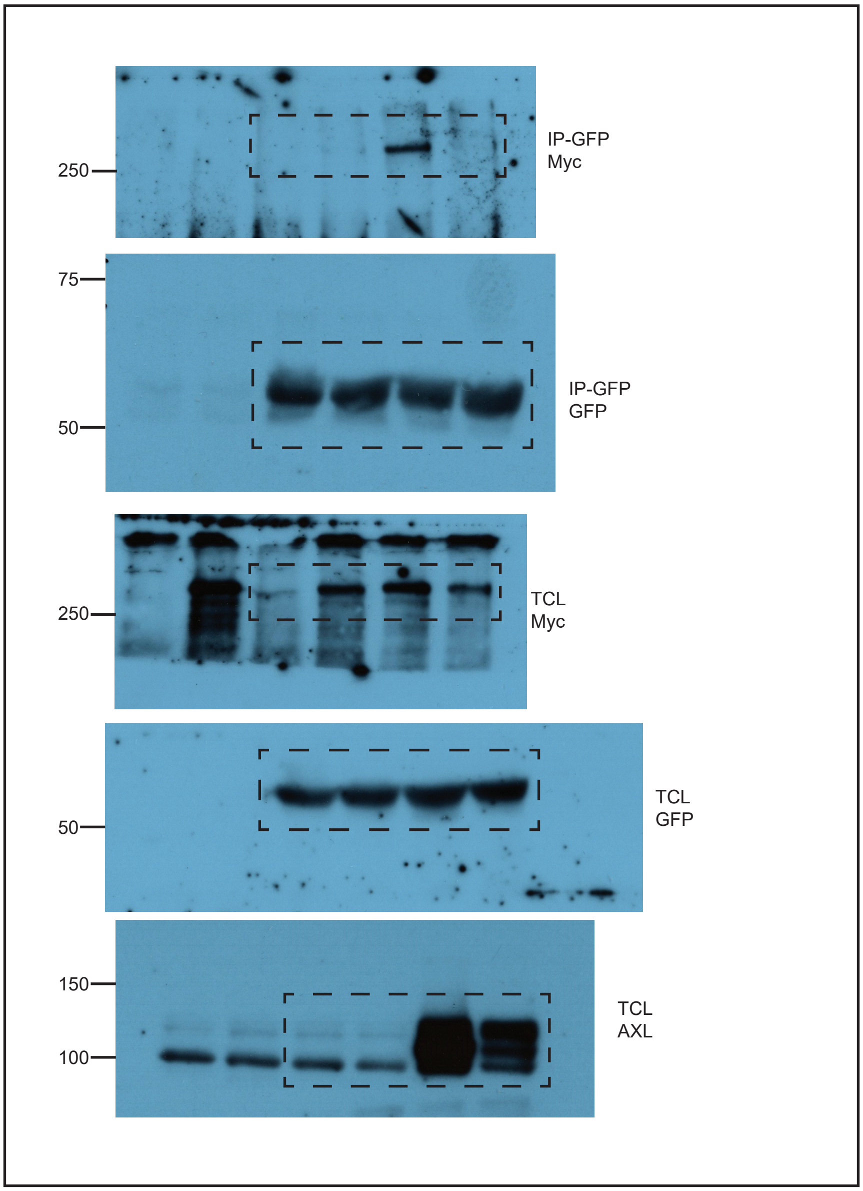

Fig 6g

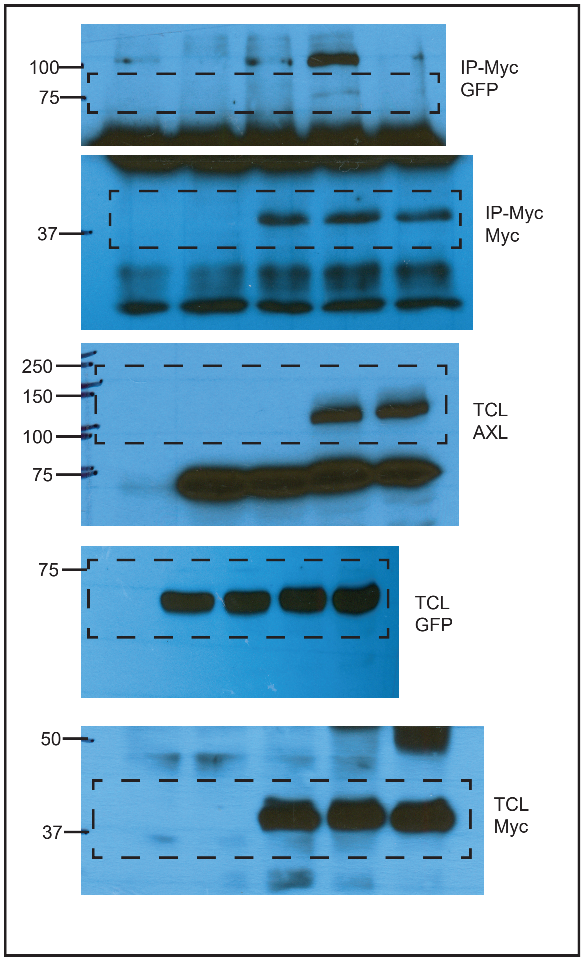

Fig 7a

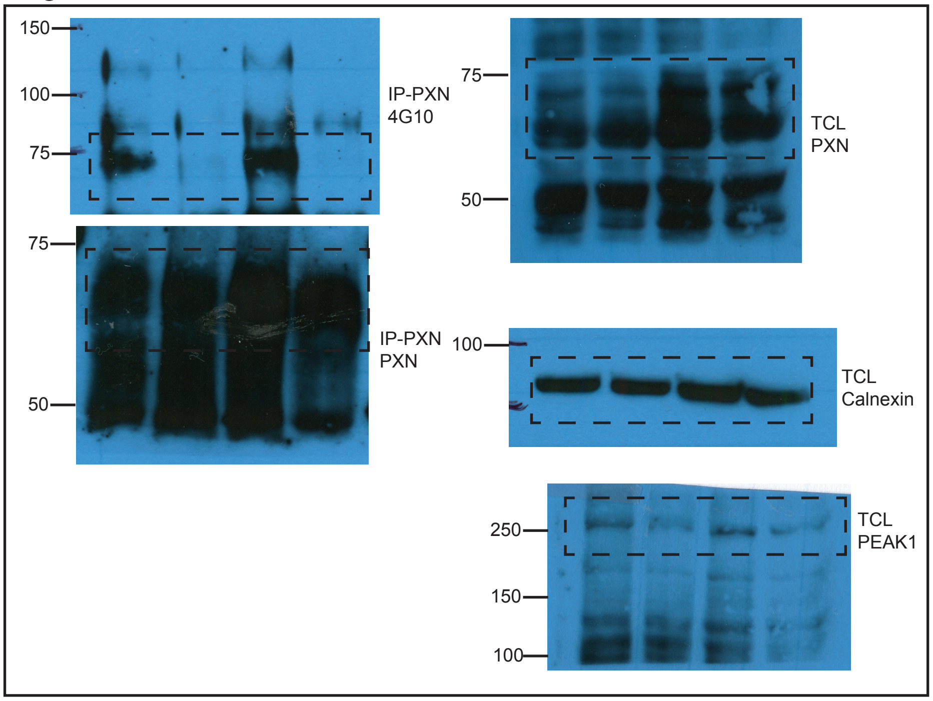

Fig 7b

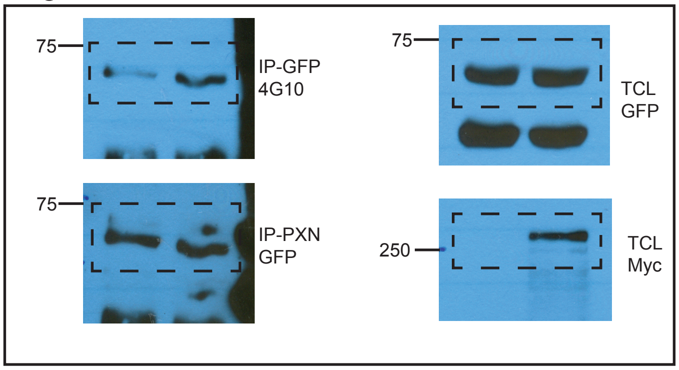

Fig 7c

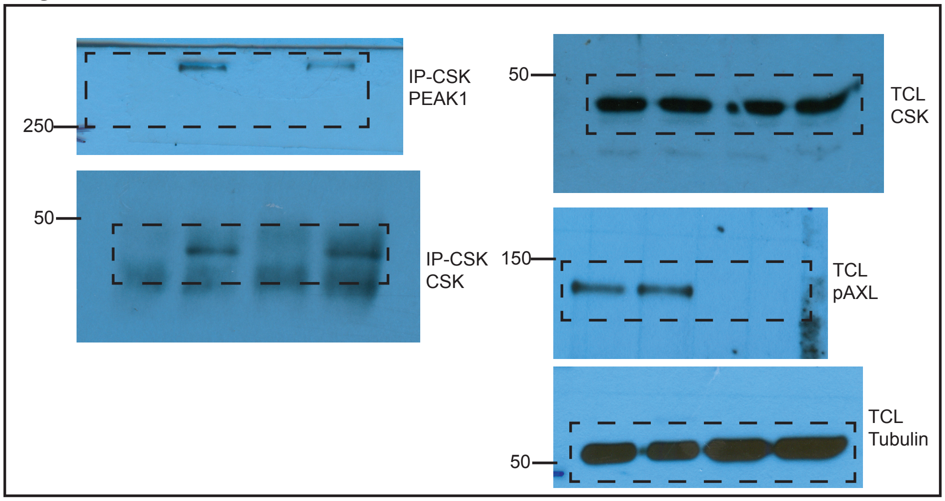

Fig 7d

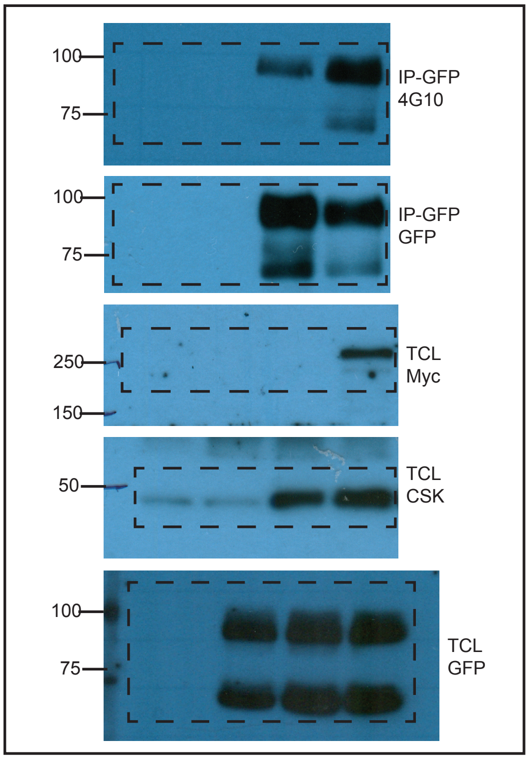

Fig 7e

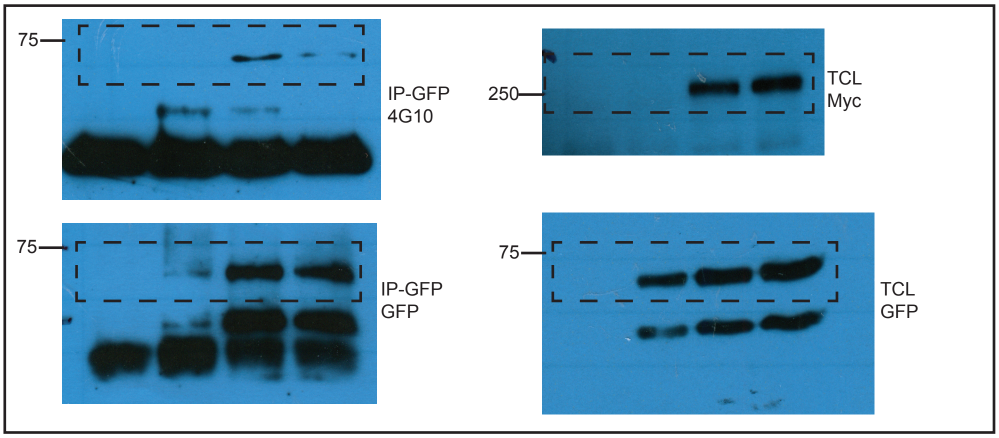

Fig 8c

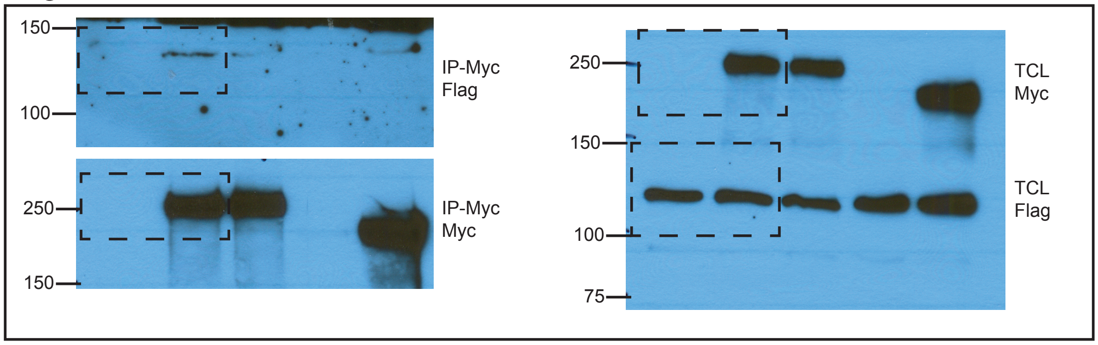
150
100
IP-Myc
Flag
250
150
IP-Myc
Myc
250
150
100
75
TCL
Myc
TCL
Flag

Fig 9a

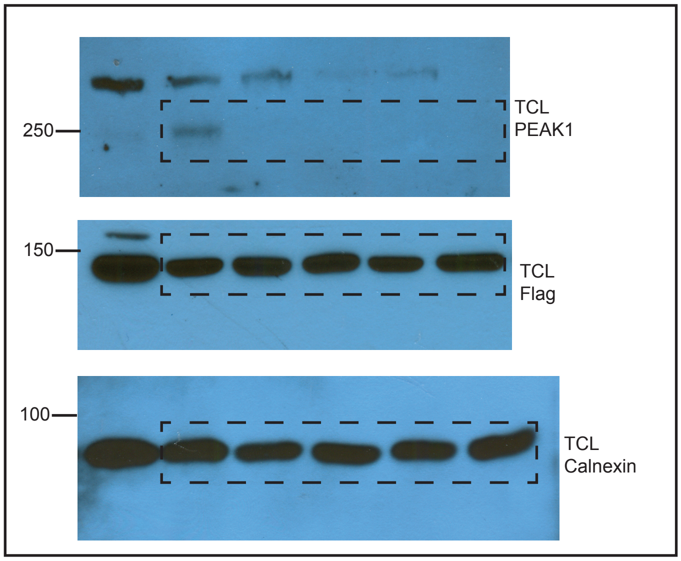

Fig 10a

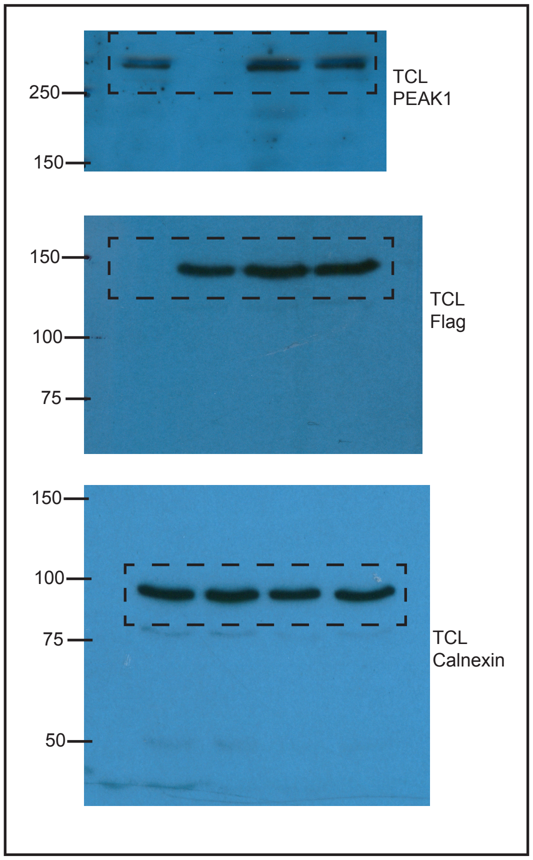

250
150
TCL
PEAK1
150
100
75
TCL
Flag
150
100
75
50
TCL
Calnexin

Fig S1a

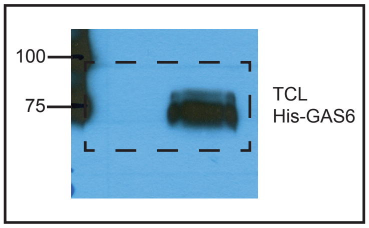

Fig S1b

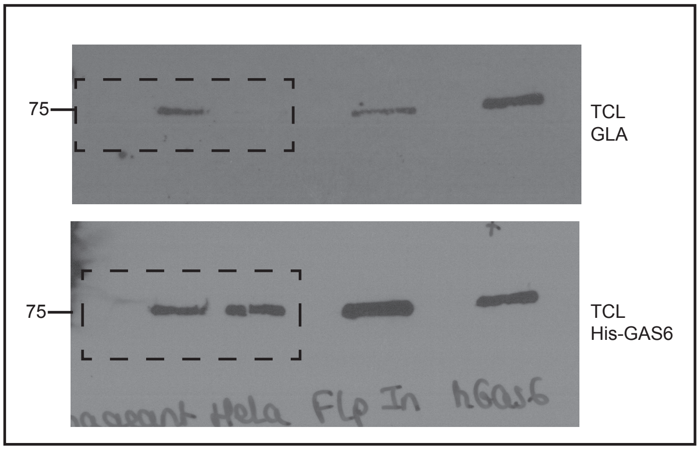

Fig S1f

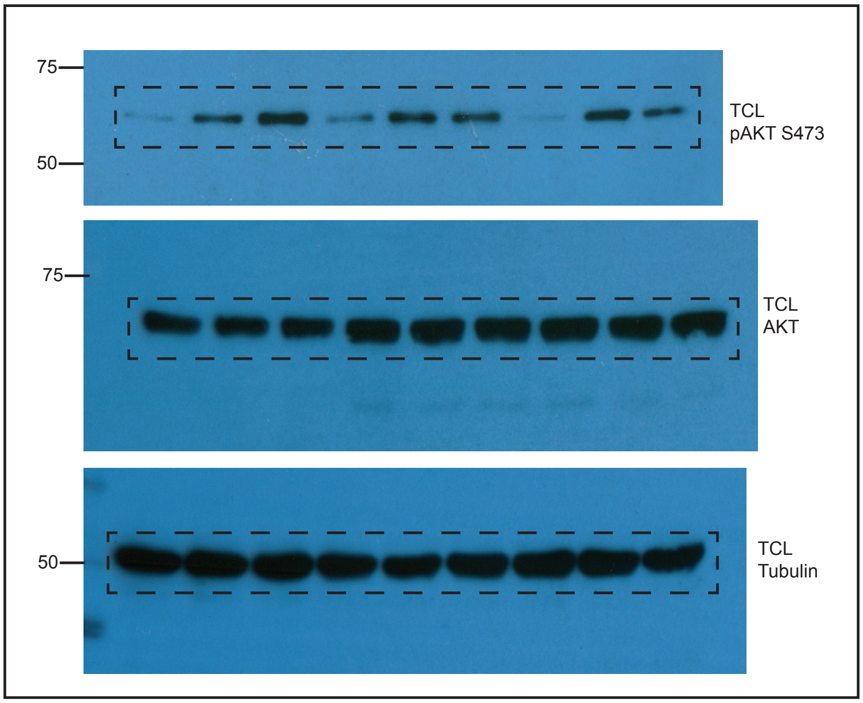

Fig S4b

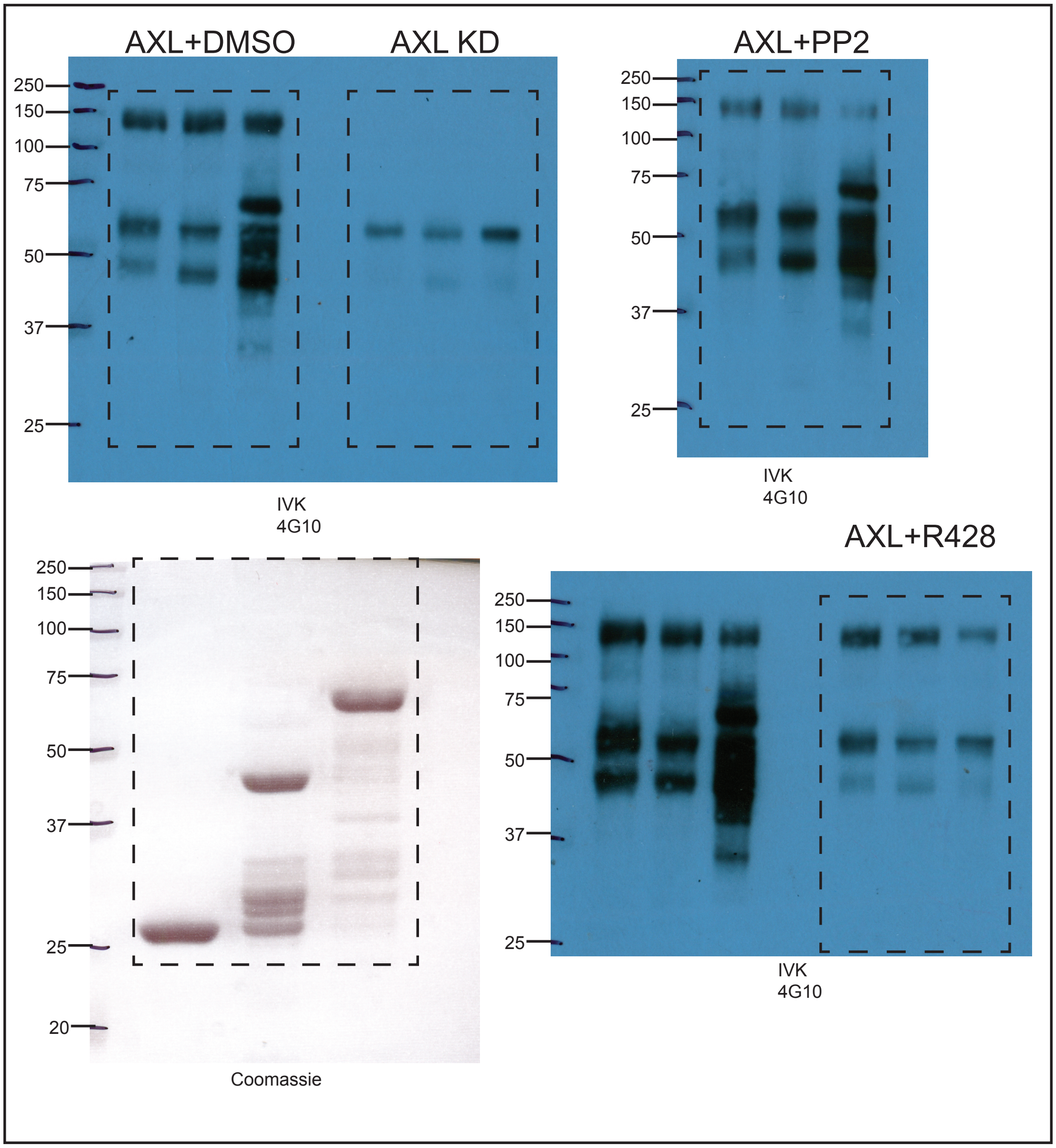

Fig S4c

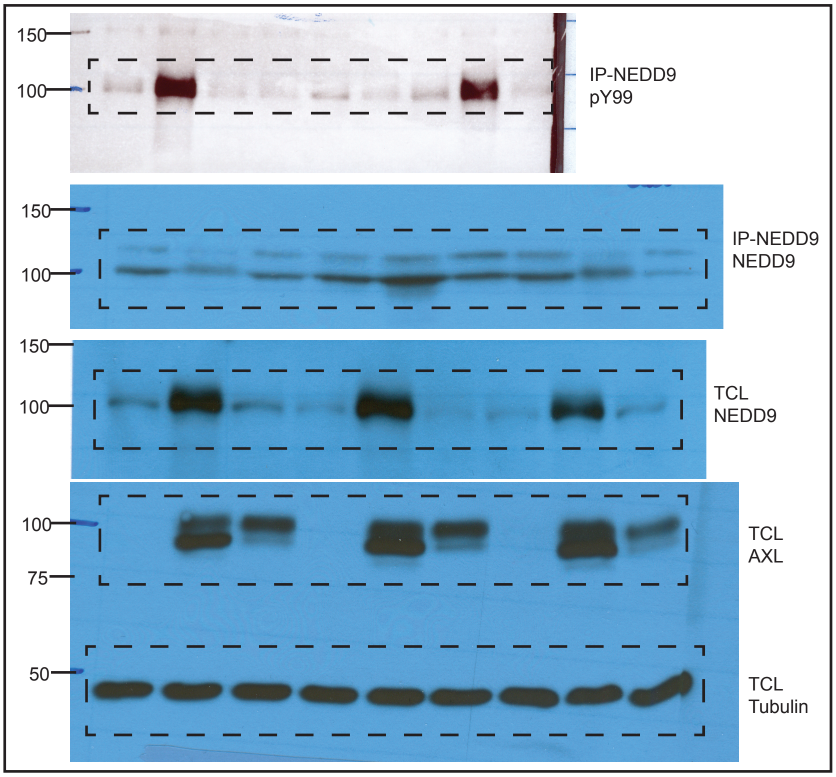

Fig S4d

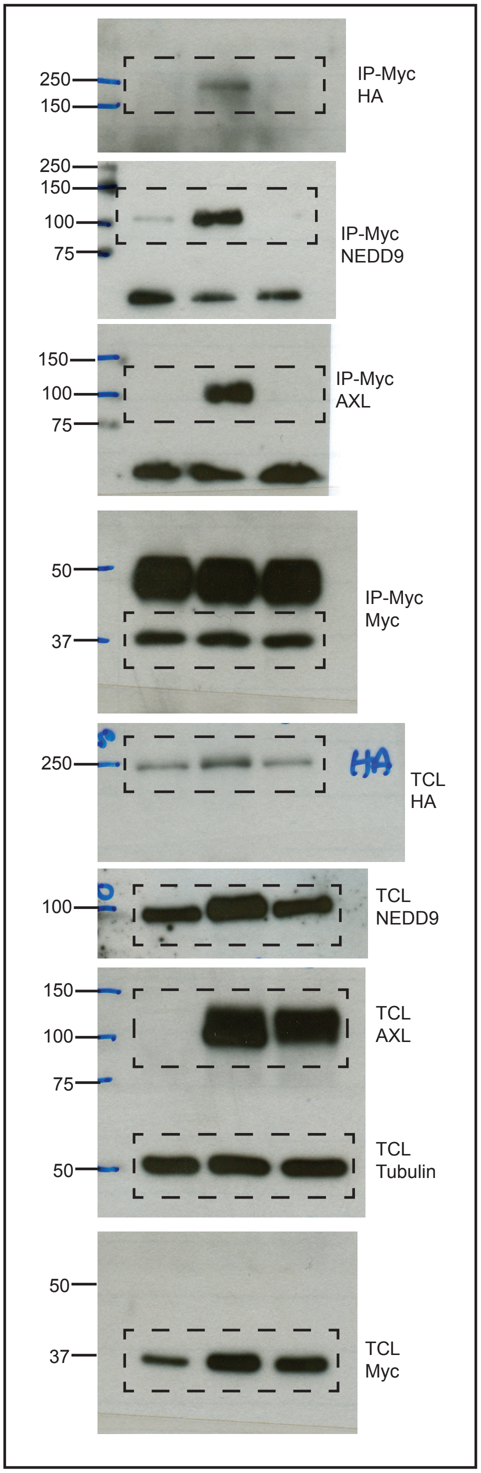

Fig S4g

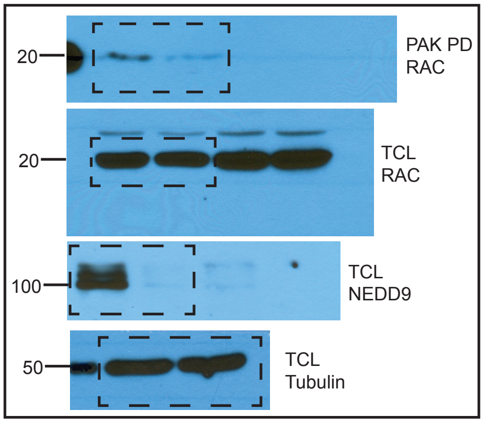

Fig S4h

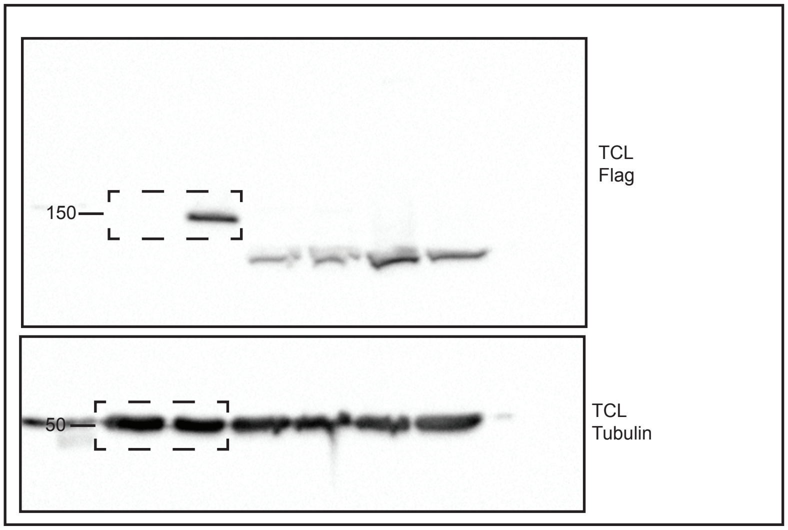

Fig S5a

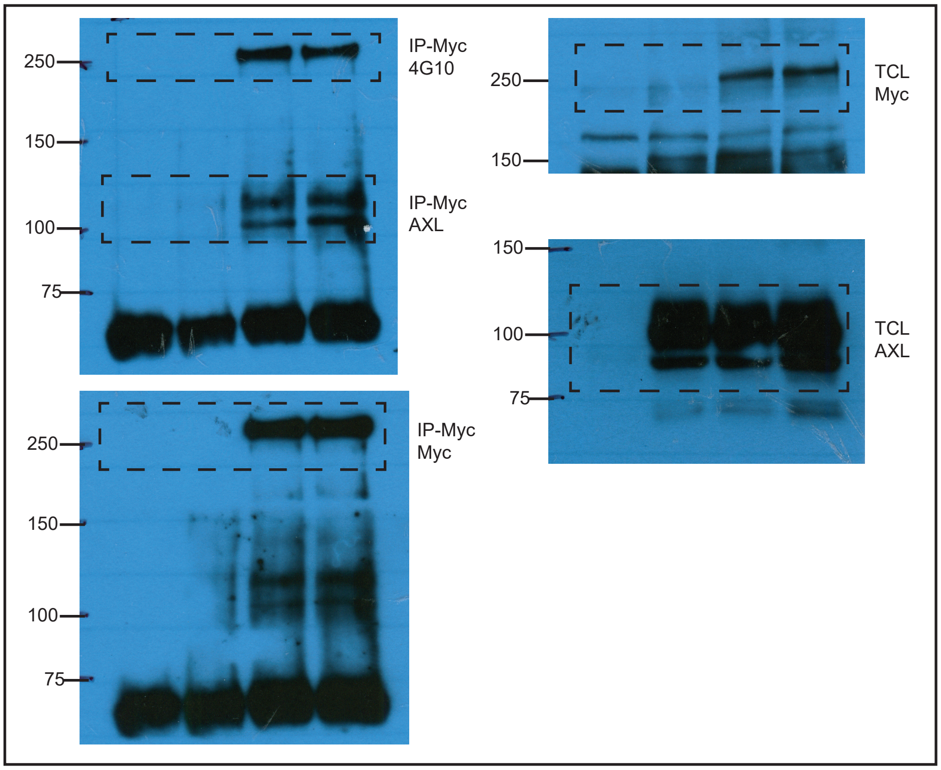

Fig S5b

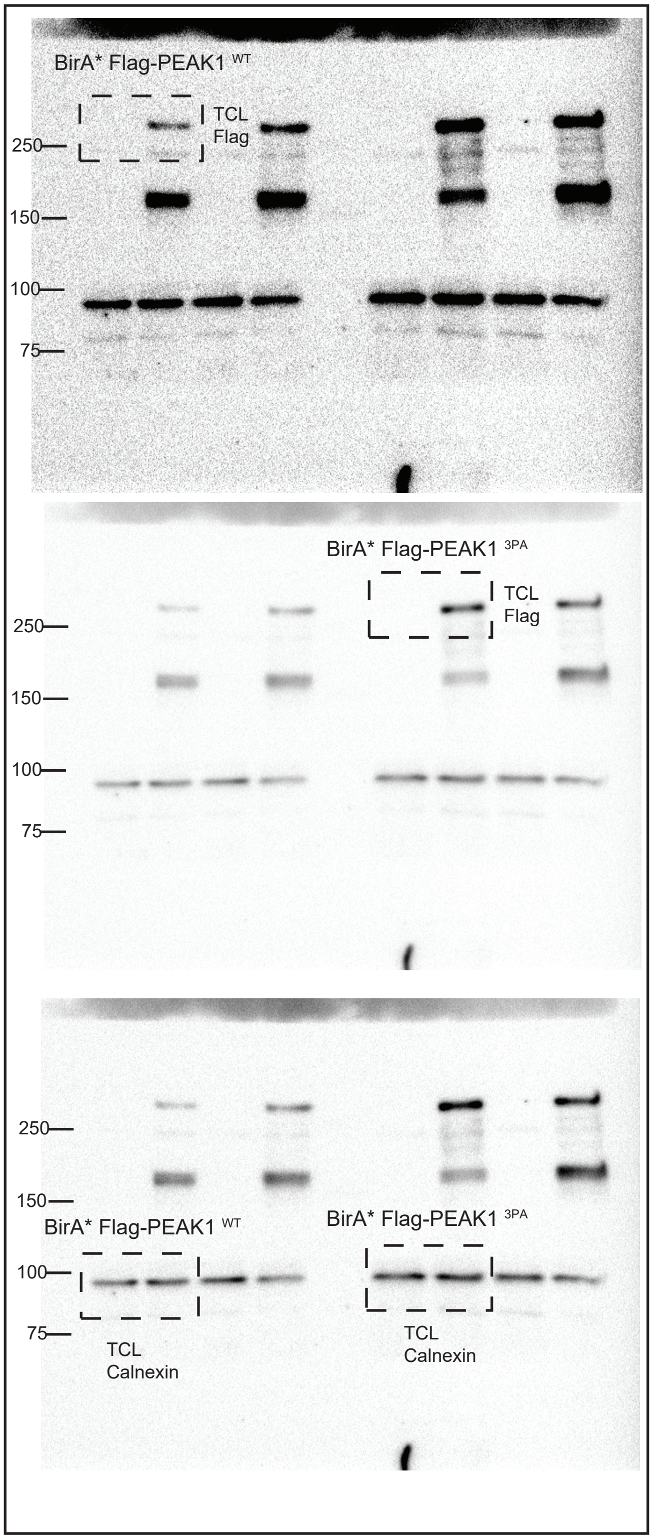

Fig S5c

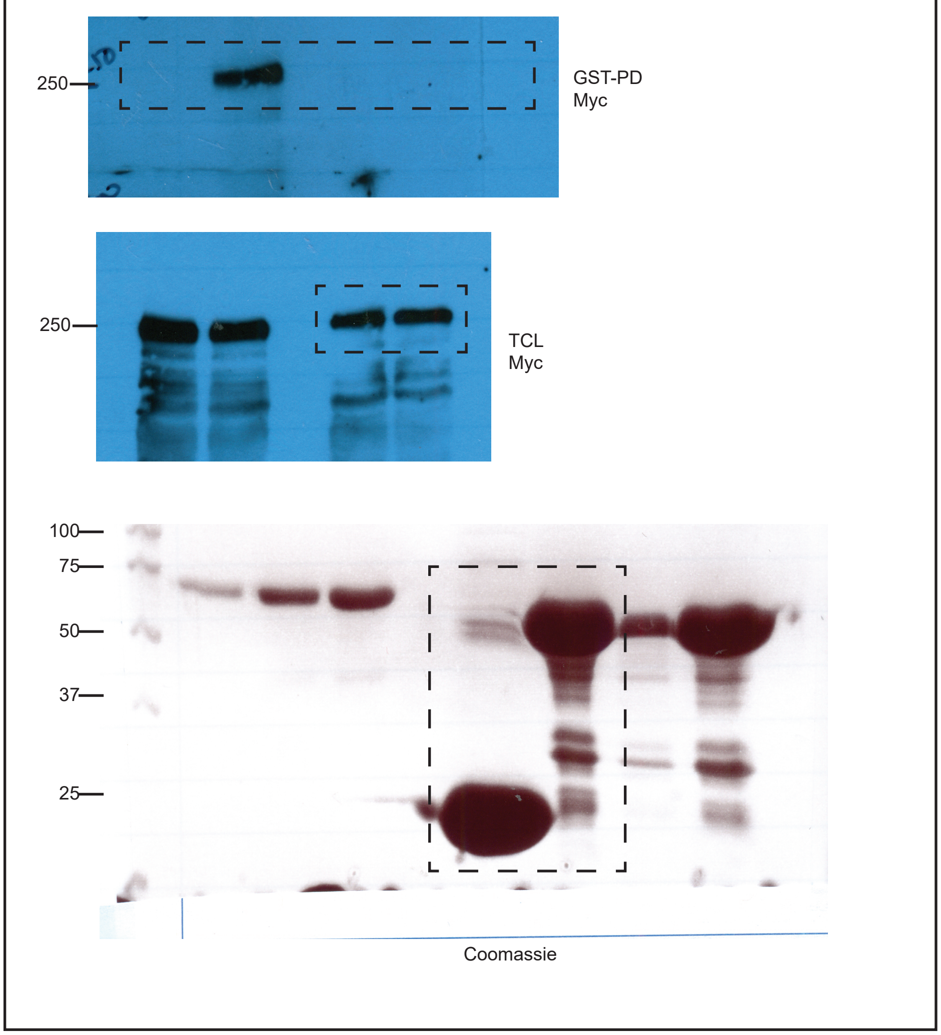

Fig S5d

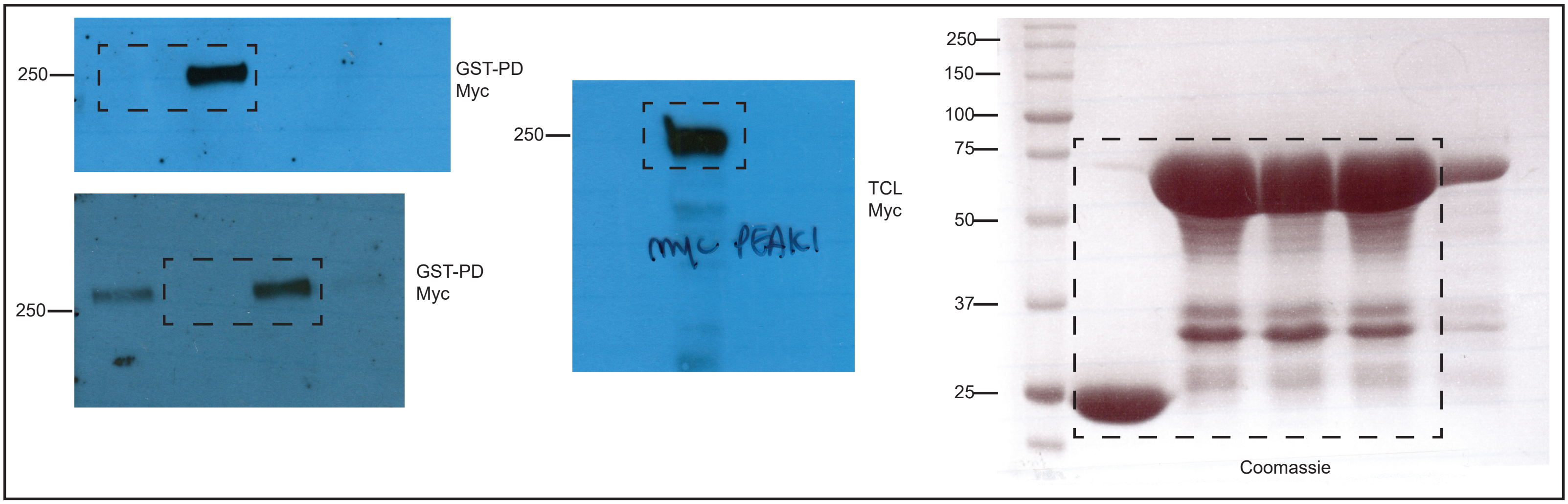

Fig S5g

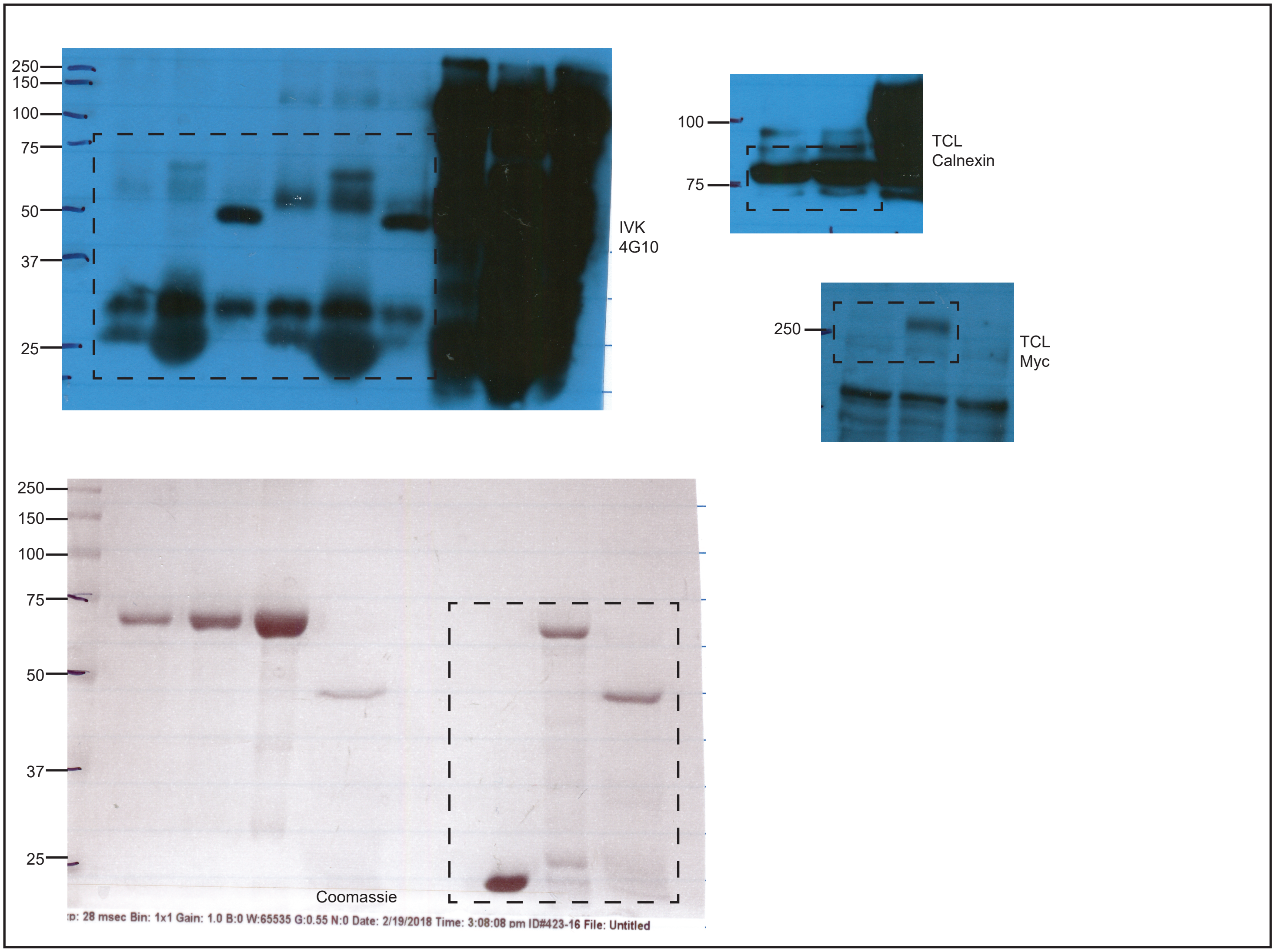

Fig S6a

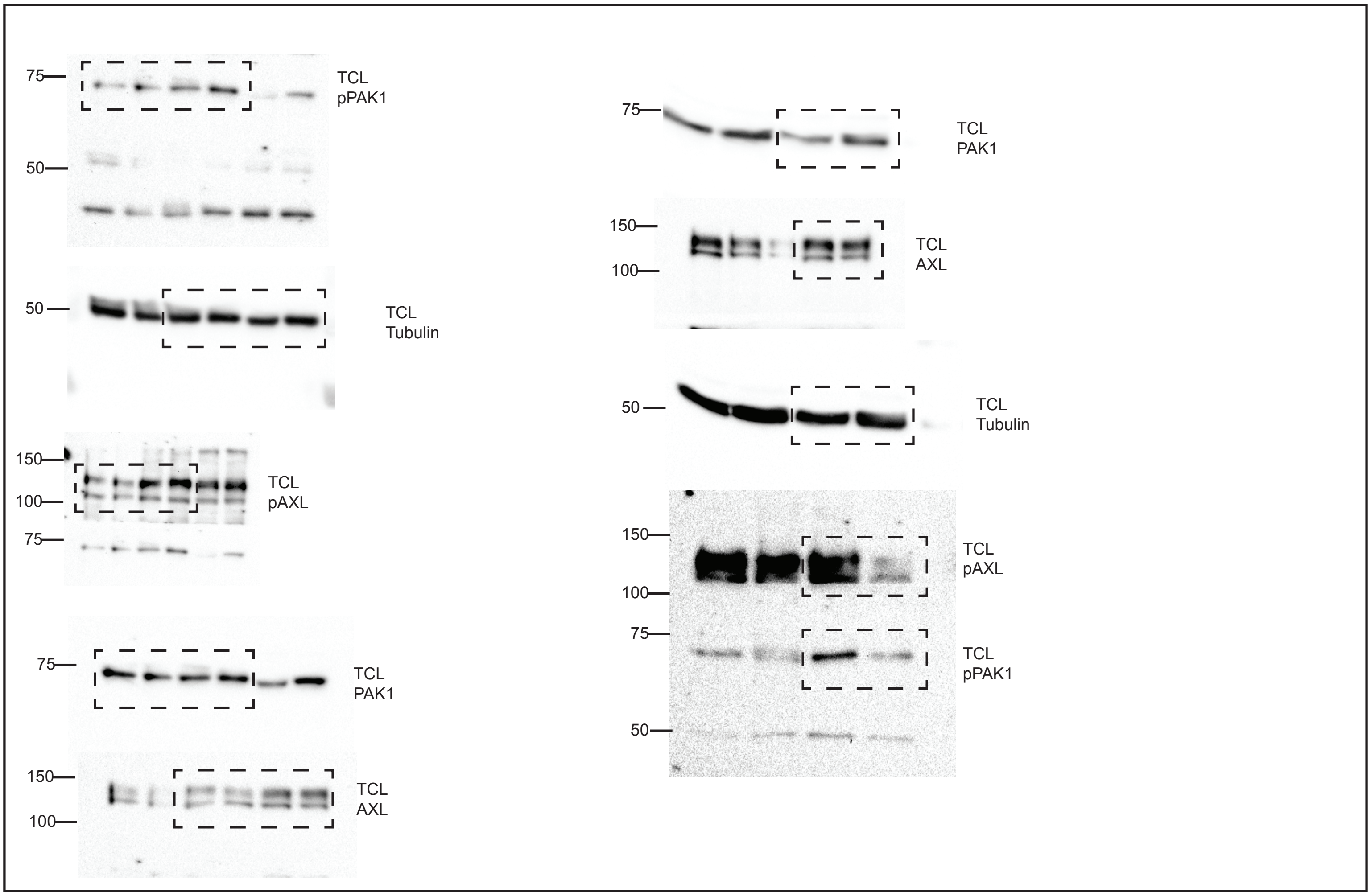

Fig S6f

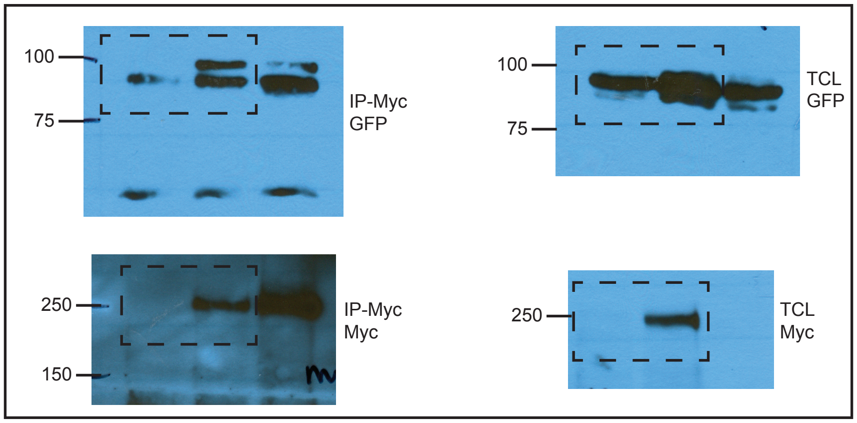

Fig S7l

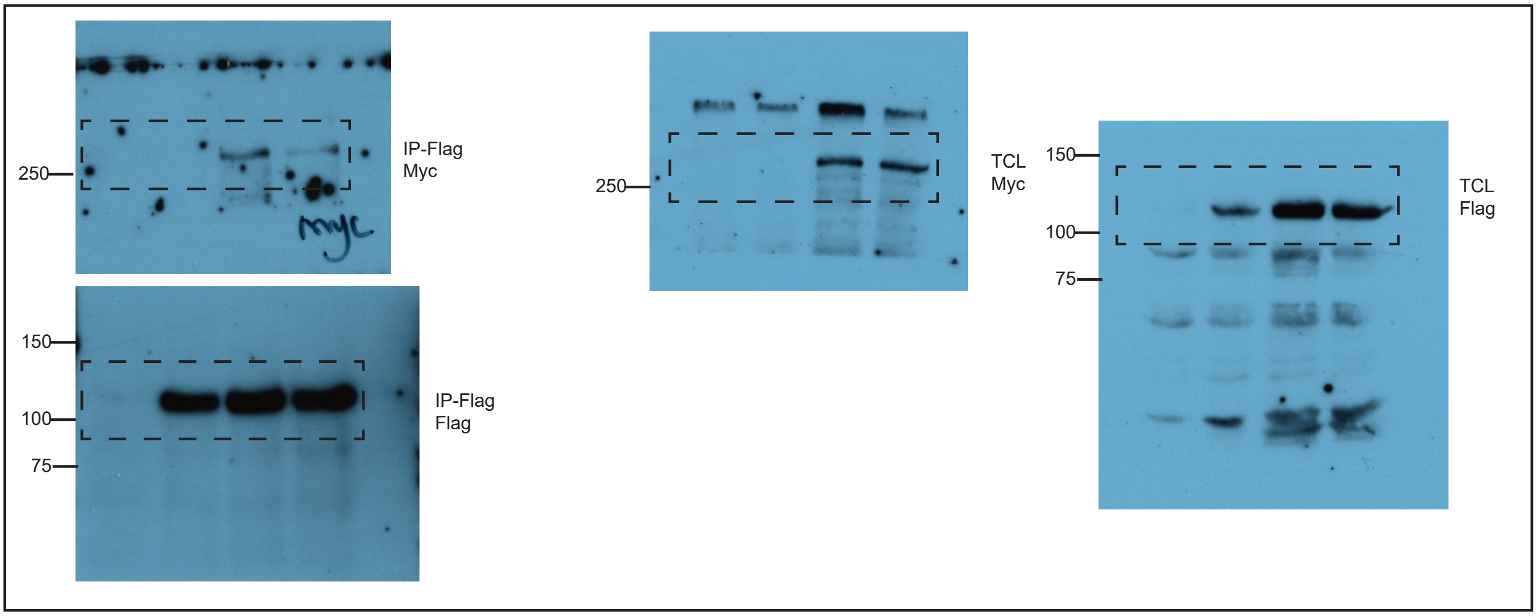

Fig S7m

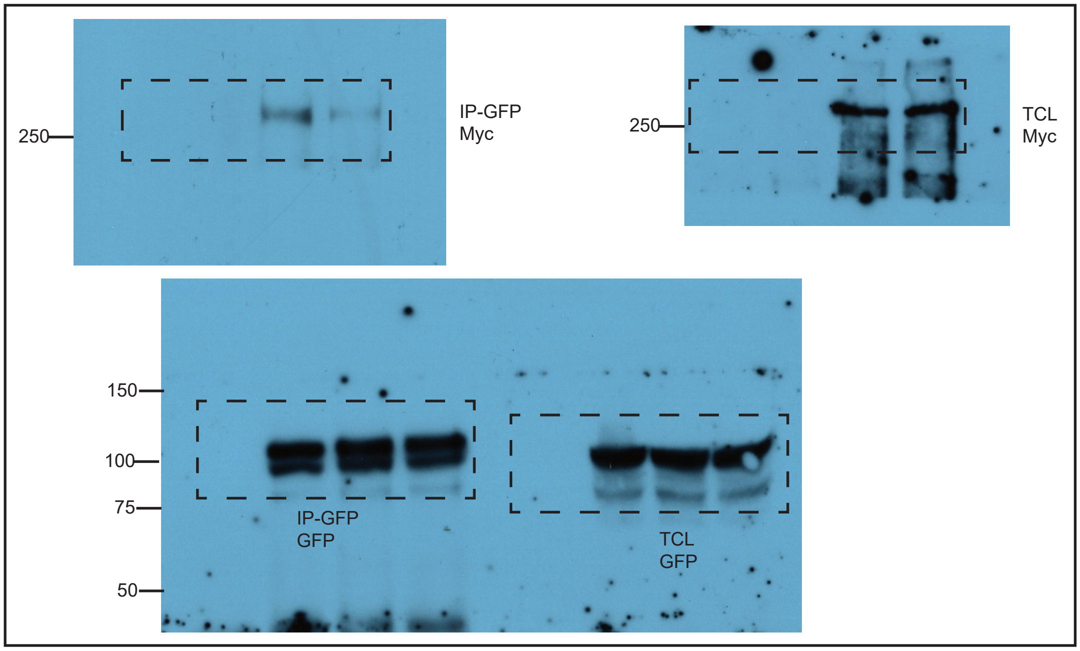

Supplementary Table S1: List of antibodies

Name of Antibody	Company	Catalog #	Purpose	Dilution
p-AXL Y702	Cell Signaling	5724	WB	1:500
p-AKT S473	Cell Signaling	9271	WB	1:1000
pPAK1 S144/pPAK2 S141	Cell Signaling	2606P	WB	1:1000
PAK1	Cell Signaling	2602	WB	1:1000
pEPHA2 Y588	Cell Signaling	12677	WB	1:500
pGSK3B S9	Abcam	ab4797	WB	1:1000
pJUN S243	Cell Signaling	2994	WB	1:500
pMYPT1 T696	Cell Signaling	5163	WB	1:500
pERK1/2 T202/Y204	Cell Signaling	4376	WB	1:1000
pCRKII Y251	Abcam	ab215751	WB	1:1000
pPXN Y118	Cell Signaling	2541	WB	1:500
pVCL Y822	Abcam	ab200825	WB	1:500
pCRMP2 T514	Cell Signaling	9397	WB	1:500
Paxillin	BD Transduction Laboratories	612405	WB	1:2000
Paxillin	BD Transduction Laboratories	612405	IF/PLA	1:200
βPix	Cell Signaling	4515	IF/PLA	1:100
GIT1	Cell Signaling	2919	PLA	1:100
GIT1	Dr. Alan Rick Horwitz	-	IF	1:100
Alexa Fluor 633 Phalloidin	Thermo Fisher Scientific	A22284	IF	5:200
Alexa 488 conjugated anti-rabbit	Thermo Fisher Scientific	A21441	IF	1:1000
Alexa 568 conjugated anti-mouse	Thermo Fisher Scientific	A11031	IF	1:1000
Alexa 488 conjugated anti-mouse	Thermo Fisher Scientific	A21200	IF	1:1000
Alexa 568 conjugated anti-rabbit	Thermo Fisher Scientific	A11011	IF	1:1000
AXL	Genescript	-	WB	1:10000
AXL	Cell Signaling	8661	PLA	1:100
pY99	Santa Cruz	sc-7020	WB	1:1000
CasL	Santa Cruz	sc-33659	WB	1:1000
Tubulin	Sigma	T5168	WB	1:10000
FAK	Santa Cruz	sc-558	IF/PLA	1:100
GFP B-2	Santa Cruz	sc-9996	WB	1:2000
GFP B-2	Santa Cruz	sc-9996	IF	1:100
4G10 HRP	EMD Millipore	16-105	WB	1:10000
HA Y-11	Santa Cruz	sc-805	WB	1:1000
c-Myc 9E10	Santa Cruz	sc-40	WB	1:2000
c-Myc A-14	Santa Cruz	sc-789	IF	1:100
Rac1	EMD Millipore	05-389	WB	1:3000
Flag M2-HRP	Sigma	A8592	WB	1:10000
Sgk269 EB-8	Santa Cruz	sc-100403	IF/PLA	1:100
PEAK1	EMD Millipore	09-274	WB	1:500
CrkII	BD Transduction Laboratories	610035	WB	1:2000
CrkII	BD Transduction Laboratories	610035	PLA	1:100
Calnexin E-10	Santa Cruz	sc-46669	WB	1:1000
CSK	Dr. Andre Veillette	-	WB	1:1000
ERK	Cell Signaling	9102	WB	1:1000

EPHA2	Santa Cruz	sc-398832	WB	1:1000
CRMP2	Santa Cruz	sc-101348	WB	1:1000
c-JUN	Santa Cruz	sc-74543	WB	1:1000
VCL	Santa Cruz	sc-73614	WB	1:1000
GSK3B	Cell Signaling	9832	WB	1:1000
MYPT1	Santa Cruz	sc-514261	WB	1:1000
GLA	Dr. Mathieu Ferron	-	WB	1:1000
Anti-Mouse HRP	Sigma	A 4416	WB	1:5000
Anti-Rabbit HRP	Santa Cruz	sc-2357	WB	1:8000
Anti-Goat HRP	Santa Cruz	sc-2020	WB	1:8000

Supplementary Table S2: List of siRNA

Name	Name	Company	Sequence
siNEDD9	siGENOME SMARTpool Human NEDD9 siRNA	Dharmacon, GE Healthcare	5’ AGGAACUGGCCUUUCGCAA 3’<br>5’ CUACCAAAAUCAGGGAAUU 3’<br>5’ CCUCUGGACUGAUGCAGCA 3’<br>5’ CCAAGAACAAGAGGUAUAU 3’
siPEAK1	siGENOME SMARTpool Human PEAK1 siRNA	Dharmacon, GE Healthcare	5’ CCAAAUCUCUCUUUACAUC 3’<br>5’ GCUAAGAGCACACCUAAGA 3’<br>5’ UAUCAAAGUUCCCAUUGUU 3’<br>5’ GAAAGACCCAUCCAUAAAG 3’
siAXL	ON-TARGETplus Human AXL siRNA	Dharmacon, GE Healthcare	5’ ACAGCGAGAUUUAUGACUA 3’<br>5’ GGUACCGGCUGGCGUAUCA 3’<br>5’ GACGAAAUCCUCUAUGUCA 3’<br>5’ GAAGGAGACCCGUUAUGGA 3’
siCRKII	siGENOME SMARTpool Human CRKII siRNA	Dharmacon, GE Healthcare	5’ GGACAGCGAAGGCAAGAGA 3’<br>5’ GAAUAGGAGAUCAAGAGUU 3’<br>5’ GGUGAGCUGGUAAAGGUUA 3’<br>5’ GGACAAGCCUGAAGAGCAG 3’
siCTRL	siGENOME Non-targeting siRNA	Dharmacon, GE Healthcare	5’ UAGCGACUAAACACAUCAA 3’
siGIT1	siGENOME SMARTpool Human GIT1 siRNA	Dharmacon, GE Healthcare	5’ GGACGACGCCAUCUAUUCA 3’<br>5’ GCACACCCAUUGACUAUGC 3’<br>5’ GGACGCCACAUCUCCAUUG 3’<br>5’ CCGCACACCCAUUGACUAU 3’
siβPIX	siGENOME SMARTpool Human βPIX siRNA	Dharmacon, GE Healthcare	5’ GGAAGAAGAUGCUCAGAUU 3’<br>5’ GAAGAGCCCUCCCAAAGGA 3’<br>5’ UCAAAGAGCUCGAGAGACA 3’<br>5’ GGAGGGCGAUGACAUUAAA 3’
siPAK1	siGENOME SMARTpool Human PAK1 siRNA	Dharmacon, GE Healthcare	5’ CAUCAAAUAUCACUAAGUC 3’<br>5’ CAACAAAGAACAAUCACUA 3’<br>5’ AGAAAUACCAGCACUAUGA 3’<br>5’ GUGAAAUGCUCUCGGCUAU 3’
siCDC42	siGENOME SMARTpool Human CDC42 siRNA	Dharmacon, GE Healthcare	5’ GGAGAACCAUAUACUCUUG 3’<br>5’ GAUUACGACCGCUGAGUUA 3’<br>5’ GAUGACCCCUCUACUAUUG 3’<br>5’ CGGAAUAUGUACCGACUGU 3’
siRAC1	siGENOME SMARTpool Human RAC1 siRNA	Dharmacon, GE Healthcare	5’ UAAGGAGAUUGGUGCUGUA 3’<br>5’ UAAAGACACGAUCGAGAAA 3’<br>5’ CGGCACCACUGUCCCAACA 3’<br>5’ AUGAAAGUGUCACGGGUAA 3’

Supplementary Table S3: List of plasmids

	Plasmids	Purpose	Origin
1	pDONR221	Backbone to generate all pDONR vectors	Addgene
2	pcDNA-hGAS6-His	Used to generate #3	Dr. Eric Manser
3	pDONR221-hGAS6-His	Used to generate #4	This study
4	pDEST-pcDNA5-hGAS6-His	Recombinant Gas6 Media	This study
5	pEGFP-NEDD9	Used to generate #9, 12, 13	Dr. Erica Golemis
6	pcDNA3.1-HA-NEDD9	Immunoprecipitation	Dr. Erica Golemis
7	pcDNA3-NEDD9	Immunoprecipitation	Dr. Michel Tremblay
8	pOG44	NEDD9 BioID	Dr. AC Gingras
9	pDONR221-NEDD9 for N-ter tag	Used to generate #11	This study
10	pcDNA5-pcDEST-BirA-Flag-N-ter	Used to generate #11, 32, 33	Dr. AC Gingras
11	pcDNA5-pDEST-BirA*-Flag-N-ter NEDD9	NEDD9 BioID	This study
12	pGEX4T1-hNEDD9 SD	NEDD9 IVK	This study
13	pGEX4T1-hNEDD9 CT	NEDD9 IVK	This study
14	pGEX4T1 GST	IVK/GST-Pulldown	JF Cote Lab
15	pCIS2-HA-DOCK3	Immunoprecipitation	Dr. David Schubert
16	pCMV-sport6-mouse AXL-WT	Immunoprecipitation	Dr. Rob Screaton
17	pCMV-sport6-mouse AXL-KD	Immunoprecipitation	JF Cote Lab
18	pcDNA3 FAK D5 HA-Tag	Immunoprecipitation	JF Cote Lab
19	pCS-3MT-6Myc	Backbone to generate all pCS-6Myc vectors	Addgene
20	pRetroX-Sgk269 WT	Used to generate #27	Dr. Roger Daly
21	pDONR221-L1-PEAK1 WT (stop)-L2	Used to generate #34, 32	This study
22	pDONR221-L1-PEAK1 Y531F (stop)-L2	Used to generate #36	This study
23	pDONR221-L1-PEAK1 3PA (stop)-L2	Used to generate #35, 33	This study
24	pcDNA5-pDEST-BirA*-Flag-N-ter PEAK1 WT	PEAK1 BioID	This study
25	pcDNA5-pDEST-BirA*-Flag-N-ter PEAK1 3PA	PEAK1 BioID	This study
26	pCS-6Myc-hPEAK1	Immunoprecipitation	This study
27	pCS-6Myc-hPEAK1 3PA	Immunoprecipitation/ Immunofluorescence	This study
28	pCS-6Myc-hPEAK1 Y531F	Immunoprecipitation	This study
29	pECFP-Flag-GIT1	Immunoprecipitation	Addgene
30	pCMV5-HA- ARHGEF7	Used to generate #41	Dr. Liliana Attisano
31	pEGFP-C2-ARHGEF7	Immunoprecipitation	This study
32	pEGFP-C2 CRKII WT	Immunoprecipitation	JF Cote Lab
33	pGEX 4T1 GST-CRKII WT	GST-Pulldown	JF Cote Lab
34	pGEX 4T1 GST-CRKII W169L	GST-Pulldown	JF Cote Lab
35	pGEX 4T1 GST-CRKII W275L	GST-Pulldown	JF Cote Lab
36	pcDNA3-Myc CRKII	Immunoprecipitation	JF Cote Lab
37	pEGFP-PAXILLIN	Live cell imaging	Addgene
38	pGEX2TK GST-PAXILLIN 1-313	Paxillin IVK	Dr. Christopher E. Turner
39	pGEX2TK GST-PAXILLIN 329-559	Paxillin IVK	Dr. Christopher E. Turner

**40**	pXM139-CSK	Immunoprecipitation	Dr. Andre Veillette
**41**	pVSV-G	Lentiviral infection	Dr. Mathieu Ferron
**42**	pRSV-Rev	Lentiviral infection	Dr. Mathieu Ferron
**43**	pMDLg/Prre	Lentiviral infection	Dr. Mathieu Ferron
**44**	pCL-Ampho	Retroviral infection	Dr. Sylvain Meloche
**45**	MSCV Luciferase PGK-hygro	Stable Luciferase expression	Addgene
**46**	LentiCas9-Blast	Stable Cas9 expression	Addgene
**47**	PEAK1 gRNA (BRDN0001145890)	Crispr PEAK1 KO1	Addgene
**48**	PEAK1 gRNA (BRDN0001147244)	Crispr PEAK1 KO2	Addgene
**49**	pRetroX-Sgk269 WT PAM mutant	Rescue of Crispr PEAK1 KO1	This study
**50**	pRetroX-Sgk269 3PA PAM mutant	Rescue of Crispr PEAK1 KO1	This study
**51**	pRetroX EV	Rescue of Crispr PEAK1 KO1	Dr. Roger Daly

Supplementary Table S4: List of primers

Oligonucleotide name	Oligonucleotide sequence	Purpose
hGas6 Forward	5’ GGGGACAAGTTTGTACAAAAAAGCAGGCTTAA TGGCCCCTTCGCTCT 3’	Gateway Cloning
hGas6 Reverse	5’ GGGGACCACTTTGTACAAGAAAGCTGGGTTTCA GTGGTGGTGGTGGTGG 3’	Gateway Cloning
hNedd9 SD Forward	5’ ATCTGGTTCCGCGTGGATCCATGCAGCAGACCT TTGGC 3’	Cloning
hNedd9 SD Reverse	5’ TCACGATGCGGCCGCTCGAGCTGAGCTGGGG AGGCTG 3’	Cloning
hNedd9 CT Forward	5’ ATCTGGTTCCGCGTGGATCCATGGATGACTACG ATTACGTCCAC 3’	Cloning
hNedd9 CT Reverse	5’ TCACGATGCGGCCGCTCGAGTGCCATCTCCAGC AAAGAGCGC 3’	Cloning
hNedd9 Forward	5’ GGGGACAAGTTTGTACAAAAAAGCAGGCTTCATG AAGTATAAGAATCTTATGG 3’	Gateway Cloning
hNedd9 Reverse	5’ GGGG AC CAC TTT GTA CAA GAA AGC TGG GTC TTAGAACGTTGCCATCTCCAGCA 3’	Gateway Cloning
GFP-PIX Forward	5’ CGAGCTCAAGCTTCGAATTCATGACCGATAATAG CAACAATCAACTGG 3’	HiFi Cloning
GFP-PIX Reverse	5’ TCAGTTATCTAGATCCGGTGGATCCTTATAGATTG GTCTCATCCCAGGCAGG 3’	HiFi Cloning
hBcar3 Forward	5’ GGGGACAAGTTTGTACAAAAAAGCAGGCTTCA TGGCTGCAGGAAAATTTGCAAG 3’	Gateway Cloning
hBcar3 Reverse	5’ GGGGACCACTTTGTACAAGAAAGCTGGGTCTC AAAGCTCTGCCTGCTTTACAGG 3’	Gateway Cloning
hPEAK1 WT Forward	5’ GGGGACAAGTTTGTACAAAAAAGCAGGCTTAATG TCTGCTTGTAACACCTTTACTGAAC 3’	Gateway Cloning
hPEAK1 WT Reverse	5’ GGGGACCACTTTGTACAAGAAAGCTGGGTTTCA GAGCGGCCGCGT 3’	Gateway Cloning
hPEAK1 ΔN Forward	5’ GGGGACAAGTTTGTACAAAAAAGCAGGCTTAAT GGTGGTCTCTTATGGACAAGG 3’	Gateway Cloning
PEAK1 Y531F Forward	5’ ATTCGATTCCAAGAAGTATGGACTTCTAGCACCA GTCCAC 3’	Mutagenesis
PEAK1 Y531F Reverse	5’ TTCTTGGAATCGAATTGCACTGGATTTTTGGAA 3’	Mutagenesis
PEAK1 3PA Forward	5’ CCTACAGCAGCCGCACTGCCAAAGAAGATGATC ATAAGAGCCAATACAGA 3’	Mutagenesis
PEAK1 3PA Reverse	5’ TGGCAGTGCGGCTGCTGTAGGTTGGGAAGCATT GGGTGC 3’	Mutagenesis
PEAK1 KO1 PAM Mutant Forward	5' TATGCTCGAAGTTCCAAAAATGCTATCAAAGTTCC CATTGTT 3'	Mutagenesis
PEAK1 KO1 PAM Mutant Reverse	5' GGAACTTCGAGCATAAGTTGGCTCGTCATGAATG ATAATT 3'	Mutagenesis
