# Supplementary material for: AXL confers cell migration and invasion by hijacking a PEAK1-regulated focal adhesion protein network
Source: Nat Commun. 2020 Jul 17;11:3586. doi: 10.1038/s41467-020-17415-x (PMC7368075; doi:10.1038/s41467-020-17415-x)
Supplement: Supplementary file 13 — Reporting Summary [file 41467_2020_17415_MOESM13_ESM.pdf]

nature research

Corresponding author(s): Jean-Francois Côté

Last updated by author(s): Jun 16, 2020

Reporting Summary

Nature Research wishes to improve the reproducibility of the work that we publish. This form provides structure for consistency and transparency in reporting. For further information on Nature Research policies, see our Editorial Policies and the Editorial Policy Checklist.

Statistics

For all statistical analyses, confirm that the following items are present in the figure legend, table legend, main text, or Methods section.

n/a	Confirmed	
☐	☒	The exact sample size (*n*) for each experimental group/condition, given as a discrete number and unit of measurement
☒	☐	A statement on whether measurements were taken from distinct samples or whether the same sample was measured repeatedly
☐	☒	The statistical test(s) used AND whether they are one- or two-sided<br>*Only common tests should be described solely by name; describe more complex techniques in the Methods section.*
☒	☐	A description of all covariates tested
☒	☐	A description of any assumptions or corrections, such as tests of normality and adjustment for multiple comparisons
☐	☒	A full description of the statistical parameters including central tendency (e.g. means) or other basic estimates (e.g. regression coefficient) AND variation (e.g. standard deviation) or associated estimates of uncertainty (e.g. confidence intervals)
☐	☒	For null hypothesis testing, the test statistic (e.g. *F*, *t*, *r*) with confidence intervals, effect sizes, degrees of freedom and *P* value noted<br>*Give P values as exact values whenever suitable.*
☒	☐	For Bayesian analysis, information on the choice of priors and Markov chain Monte Carlo settings
☒	☐	For hierarchical and complex designs, identification of the appropriate level for tests and full reporting of outcomes
☒	☐	Estimates of effect sizes (e.g. Cohen's *d*, Pearson's *r*), indicating how they were calculated

Our web collection on statistics for biologists contains articles on many of the points above.

Software and code

Policy information about availability of computer code

Data collection	Phosphoprotein identifications and phospho-modulation analyses were performed using MaxQuant (version 1.6.1.0) against the human RefSeq protein database (version July 29, 2016). ProHits software (version 6.02) was used to collect the raw BioID mass spectrometry data. Microscopy pictures were collected with commercially available softwares: ZenBlack 2009 for confocal and ZenBlue for spinning disk microscopy from Carl Zeiss MetaExpress (version 3.1.0.97).Videos and images were obtained using IMARIS 8.0.
Data analysis	Mass spectrometry data was analyzed using the tool integrated in ProHits (version 6.0.2). Interactomes were generated using Cytoscape (version 3.7.0). Dotplots of over-represented KEGG pathways (pvalueCutoff = 0.05) were generated with the ClusterProfiler package in R (version 3.14.3) (www.rproject.org). Immunofluorescence images were analyzed using Fiji (version 2.0.0-rc69/1.52p). All statistics were analyzed with Prism 6 GraphPad (version 6.0) and Microsoft Excel (version 16.9.1).

For manuscripts utilizing custom algorithms or software that are central to the research but not yet described in published literature, software must be made available to editors and reviewers. We strongly encourage code deposition in a community repository (e.g. GitHub). See the Nature Research guidelines for submitting code & software for further information.

Data

Policy information about availability of data

All manuscripts must include a data availability statement. This statement should provide the following information, where applicable:

- Accession codes, unique identifiers, or web links for publicly available datasets
- A list of figures that have associated raw data
- A description of any restrictions on data availability

The raw proteomics data, which are presented in Fig. 1, 2, 4, and 5 are uploaded to the MassIVE archive [doi:10.25345/C5MD88]. The source data underlying Figs

3b, e, g-h, 4e, 5e, g, 6f, 7g-h, 8b, e, g, 9c, e, g and 10c-e, g-h and Supplementary Figs 3c-e and 4e-f, 6c-e, h and 7a-c, e-j are provided as a Source Data file. The raw western blots underlying Figs 1b, e, 4a-c, f, 5b-c, 6b-d, g, 7a-e, 8c, 9a and 10a and Supplementary Figs 1a-b, f, 4b-d, g-h, 5a-d, g, 6a, f and 7l-m are provided as a supplementary figure in Supplementary Information file. All databases used in this study are mentioned in the methods section under Bioinformatics analysis. All cell lines and constructs generated in the manuscript are available upon requests from authors.

All proteomics data were imported into a local MySQL database (server 5.7). Published protein-protein interaction databases were downloaded from BioGRID (human version3.4.164), Uniprot (human release 2017-10), CORUM (version 3.0relsease 03-09-2018), Gene Ontology Annotation Database (GOA) and Pfam (human version 32.0). The application GeneMANIA (version 3.4.1 and its human database version 13 July 2017) or the builtin PSICQUIC client was used to generate protein-protein networks by merging networks generated from Intact, Mint, Reactome and Uniprot databases in Cytoscape. Each protein was annotated in Cytoscape by importing the Gene Ontology Annotation Database (GOA) and our phospho-modulation values, in order to identify relevant phospho-modulated functions. We also identified phosphatases and kinases families by importing the Phosphatome and Kinome classifications. Functional enrichment analyses of identified interactors were assessed with g:Profiler against the Gene Ontology and KEGG databases with moderate hierarchical filtering and by applying statistical correction with the Benjamini-Hochberg FDR method.

Field-specific reporting

Please select the one below that is the best fit for your research. If you are not sure, read the appropriate sections before making your selection.

☒ Life sciences ☐ Behavioural & social sciences ☐ Ecological, evolutionary & environmental sciences

For a reference copy of the document with all sections, see nature.com/documents/nr-reporting-summary-flat.pdf

Life sciences study design

All studies must disclose on these points even when the disclosure is negative.

Sample size	The study aimed to systematically characterize the phosphoproteome of AXL kinase. We dictated the number of GAS6 ligand stimulation points to three (5, 10 and 20min) used for mass spectrometry (see text for details). Since SILAC approach for phosphoproteomics is a quantitative method in comparison to other phosphoproteomic methods, we chose to perform our phosphoproteomic screens only in two replicates, since previously published manuscripts have used SILAC approach for proteomics in one replicate and was sufficient to be published.<br>The BioID baits were chosen based on their phosphomodulation by AXL kinase and that were found to be significantly modulated in the two replicates of the phosphoproteomic screen. No statistical method was used to pre-determine sample size in other methods. BioID proteomics was performed in duplicates for each bait and each control.
Data exclusions	No data was excluded in this study.
Replication	Almost all experiments were replicated at least 3 times with similar results. The SILAC experiments were performed in two replicates. The number of time an experiment was replicated is explicitly mentioned in the figure legends.
Randomization	Mass spectrometry data was, as much as possible, randomly acquired and at different time to mitigate batch effects. Other in vitro experiments were also randomized in a sense where they were done at different times and different biological samples.
Blinding	Unbiased methods were used to analyze images whenever possible. In addition, blinding was used during data collection and analysis of all mass spectrometry data. However, during analysis, blinding was not performed when choosing a set of pathways and proteins we had significant differences for, and hence were further the focus of the rest of the study.

Reporting for specific materials, systems and methods

We require information from authors about some types of materials, experimental systems and methods used in many studies. Here, indicate whether each material, system or method listed is relevant to your study. If you are not sure if a list item applies to your research, read the appropriate section before selecting a response.

Materials & experimental systems

n/a	Involved in the study	
☐	☒	Antibodies
☐	☒	Eukaryotic cell lines
☒	☐	Palaeontology and archaeology
☐	☒	Animals and other organisms
☒	☐	Human research participants
☒	☐	Clinical data
☒	☐	Dual use research of concern

Methods

n/a	Involved in the study	
☒	☐	ChIP-seq
☒	☐	Flow cytometry
☒	☐	MRI-based neuroimaging

Antibodies

Antibodies used	All antibodies used in this study are described in the Method section. See Supplementary Table 1 for information : Antibody name

	and clone number (where available), Source, Catalog number, Dilutions that were used, Incubation times, and applications).
Validation	Almost all antibodies used in this study are commercially obtained and were validated by the commercial suppliers (see specific websites for full information). The website notes validations statements for western blot antibodies such as the following: This Anti-PEAK1 Antibody is validated for use in WB for the detection of PEAK1. For other purposes such as immunoprecipitations and immunofluorescence we validated the antibodies in human cell lines and when possible, generate a knockdown of the gene by siRNA to validate the specificity of the antibody further, when more than one band is present.

Eukaryotic cell lines

Policy information about cell lines

Cell line source(s)	Flp-In T-REx HEK293 were obtained from Thermo Fisher. MDA-MB-231, Hs578T, HEK293T (293T), and HeLa cells were obtained from ATCC.
Authentication	None of the cell lines were authenticated. The Flp-In T-REx cell lines were validated by the fact that they (1) recombined cDNAs in their genomes when transfected with pOG44 and (2) they showed the expected tetracycline-dependent expression of the desired proteins. The MDA-MB-231 PEAK1 KO and rescued cells were validated via western blotting.
Mycoplasma contamination	The cell lines were not routinely tested for mycoplasma contamination.
Commonly misidentified lines (See ICLAC register)	None of the cell lines used in this study are listed in the ICLAC database.

Animals and other organisms

Policy information about studies involving animals; ARRIVE guidelines recommended for reporting animal research

Laboratory animals	Tail veins and graft experiments were conducted in athymic nude NU/J mice obtained from Jackson Laboratories in a SPF facility. All mice obtained were females. For mammary fat pad grafts, 3 weeks old mice were used. For experimental metastasis assay, 6-8 weeks old mice were used.
Wild animals	Study did not involve wild animals
Field-collected samples	Study did not involve samples from the field
Ethics oversight	All animal experiments were approved by the Animal Care Committee of the Institut de Recherches Cliniques de Montréal and complied with the Canadian Council of Animal Care guidelines.

Note that full information on the approval of the study protocol must also be provided in the manuscript.
